# A transcriptomic map of ligand-receptor interactions within bovine antral follicles predicts intrafollicular factors for improving oocyte quality

**DOI:** 10.1186/s40104-026-01469-w

**Published:** 2026-07-14

**Authors:** Zhaochen Wang, Zhenni Zhang, Yawen Tang, Jian Cui, Meiqiang Chu, Jianhui Tian, Yinjuan Wang, Lei An

**Affiliations:** 1 Frontiers Science Center for Molecular Design Breeding (MOE), China Agricultural University, Beijing, 100193 P.R. China; 2 Key Laboratory of Animal Genetics, Breeding and Reproduction, of the Ministry of Agriculture and Rural Affairs, China Agricultural University, Beijing, 100193 P.R. China; 3 State Key Laboratory of Animal Biotech Breeding, China Agricultural University, Beijing, 100193 P.R. China; 4 National Engineering Laboratory for Animal Breeding, China Agricultural University, Beijing, 100193 P.R. China; 5 College of Animal Science and Technology, China Agricultural University, Beijing, 100193 P.R. China; 6 College of Agriculture and Forestry Science, Linyi University, Linyi, Shandong Province 276000 P.R. China

**Keywords:** Antral follicle, Intercellular communication, IVM, Ligand-receptor interaction, MGCs

## Abstract

**Background:**

The ovarian follicle is an enclosed microenvironment that ensures oocyte growth and maturation, making it essential for female fertility. Normal folliculogenesis development and oocyte maturation strictly rely on orchestrated intercellular interactions among the mural granulosa cells (MGCs), cumulus cells (CCs), and the oocyte within antral follicles. Despite the importance, how these cell populations communicate remains largely unknown. Due to this knowledge gap, current oocyte in vitro maturation (IVM) systems fail to fully replicate the physiological follicular microenvironment due to the lack of MGC regulation, leading to poor oocyte quality and lower success rates of assisted reproductive technologies.

**Results:**

RNA-seq was performed on MGCs, CCs, and oocytes isolated from single bovine antral follicles at different developmental stages (3–5 mm, 6–8 mm and 9–12 mm). Using a ligand-receptor interaction scoring strategy, we constructed a dynamic intrafollicular interaction map. We revealed that MGCs are the most active cell type, acting as either secretors or receivers, based on the number of ligands and receptors it expresses, as well as the number of interaction pairs it establishes with other cell types. Intriguingly, intrafollicular crosstalk exhibits spatiotemporal commonality, characterized by shared ligand-receptor usage and potential functional redundancy across cell types and stages. Notably, the intrafollicular communication map enables the prediction of MGC-secreted factors that can improve the oocyte quality after IVM. Based on the predicted candidate factors, we have confirmed that supplementation with pleiotrophin (PTN) significantly improved in vitro matured oocyte quality and subsequent embryonic development. In addition, we also revealed significant heterogeneity at single-follicle resolution, unhealthy follicles showed aberrant activation or loss of partial core interaction signaling, supporting the hypothesis that these interactions play a critical role in determining follicular fate.

**Conclusions:**

By reconstructing a map of intrafollicular interactions, our study provides novel insights and important clues for understanding cellular and molecular mechanisms that fine-tune antral folliculogenesis. We also offer a roadmap for predicting candidate factors that can be used for optimizing mammalian in vitro embryo production systems.

**Supplementary Information:**

The online version contains supplementary material available at 10.1186/s40104-026-01469-w.

## Introduction

The normal development of ovarian follicles is extremely critical for maintaining fertility in female mammals [1]. During the successive stages of follicular development, a complex and dynamic relationship between the oocyte and the antral follicle housing it is of prime importance for oocyte growth and maturation [2]. The antral follicle is a highly organized microenvironment shaped by coordinated interactions among multiple somatic and germ cells, including the theca cells, mural granulosa cells (MGCs), cumulus cells (CCs), and the oocyte. Theca cells form the outer follicular layer, whereas MGCs lie adjacent to the basement membrane separating the granulosa and theca compartments. CCs closely surround the oocyte and, together with the oocyte itself, form a functional unit at the center of the follicle. These spatially distinct cell populations communicate intensively to regulate follicular growth, oocyte maturation, and steroidogenic function [3, 4].

These intrafollicular cell populations form a highly sophisticated regulatory network that enables multidimensional communication within the follicle [5, 6]. For instance, MGCs, together with theca cells as the principal gonadotropin-responsive somatic compartments, contribute to hormonal synthesis and follicular homeostasis, while MGCs also serve as a key conduit for nutrient transport can precisely regulate the oocyte’s meiotic progression by controlling intrafollicular cGMP signaling, thereby ensuring adequate cytoplasmic accumulation during the prolonged growth phase [7–11]. Conversely, oocyte-derived paracrine factors, such as growth differentiation factor 9 (GDF9) and bone morphogenetic protein 15 (BMP15), act as "retrograde regulatory signals" that are essential for the proliferation and differentiation of granulosa cells during early preantral stages [12, 13]. In antral follicles, they primarily target the adjacent cumulus cells to promote cumulus expansion, regulate metabolic cooperativity, and maintain the distinct cumulus phenotype, thereby establishing a closed loop of intercellular communication [14, 15]. Despite the importance, there remains a significant lack of comprehensive understanding regarding how three cell types dynamically "communicate" with each other, or even with themselves, across developmental stages. This knowledge gap substantially limits our deeper insight into the fundamental mechanisms of follicular development.

Elucidating the cellular interaction mechanisms within the antral follicular microenvironment is of urgent practical importance for overcoming the technical bottlenecks in in vitro maturation (IVM) of oocytes, a core component of the in vitro embryo production. The quality of oocytes matured in vitro has consistently failed to match that of their in vivo counterparts, and this failure has been thought to be the main factor affecting blastocyst yields [16]. The root cause of these limitations lies in the fundamental disparity between the in vitro culture system and the physiological follicular microenvironment [17, 18]. Compared with in vivo maturation during which the oocyte relies on a closed regulatory network formed by MGC–CC–oocyte crosstalk, conventional IVM systems only culture cumulus-oocyte complexes (COCs), completely lacking the physical connection and functional regulation afforded by MGCs. As a result, current IVM systems are unable to mimic the intrafollicular communication present in vivo, leading to insufficient oocyte maturation. Therefore, systematically deciphering the interactive mechanisms between MGCs and COCs, and identifying key regulatory molecules derived from MGCs, represent critical scientific steps toward enhancing the efficiency of IVM and improving oocyte quality.

In this study, we utilized the cattle antral follicle as a model system, as cattle are a representative species that typically ovulate a single dominant follicle per cycle. By isolating MGCs, CCs, and oocytes from follicles at different developmental stages, we constructed a dynamic map of intrafollicular dialogue among these three cell types during antral follicular development. Our analysis revealed that MGCs exhibit the most pronounced differential gene expression patterns and contain the highest number of ligand-receptor pairs. Further interaction analysis indicated that bovine antral follicles exhibit a spatiotemporally specific communication commonality, accompanied by a potentially overlapping ligand-receptor usage pattern. This study not only provides novel insights into the cellular interaction mechanisms within bovine antral follicles but also offers a theoretical foundation and potential technical strategies for optimizing livestock IVM systems by mimicking the in vivo microenvironment. Furthermore, these findings may serve as a valuable reference for improving in vitro oocyte maturation techniques in human assisted reproductive technologies.

## Materials and methods

All animal studies were conducted in accordance with the China Agricultural University Institutional Animal Care and Use Committee.

### Collection of MGCs, CCs and oocytes

Ovaries from *Bos taurus* cows were obtained at a local slaughterhouse and transported to the laboratory within 3 h in insulated containers maintained at 30–35 °C [19]. Upon arrival, ovaries were rinsed in pre-warmed normal saline supplemented with 100 IU/L penicillin and 50 mg/L streptomycin. Using sterile scalpel blades, excess stromal tissue was removed, and visible antral follicles were exposed. In 0.1% PVA-PBS, peri-follicular connective tissue was carefully trimmed (Fig. S1A), and follicles were categorized by diameter into three size classes: 3–5 mm, 6–8 mm, and 9–12 mm (Fig. S1B). Standard IVM procedures typically utilize oocytes arrested at the germinal vesicle (GV) stage, retrieved from follicles with diameters below 8 mm [20, 21]. Large preovulatory follicles (14–18 mm) were excluded because those follicles may be dominated by luteinization and ovulatory signaling and are likely preparing for the LH surge, representing a physiologically distinct stage that falls outside the scope of our current investigation. To ensure robust biological replication and minimize potential heterogeneity caused by prolonged processing times, sample collection was conducted across 3–4 independent batches. In each batch, only 1–2 ovaries were processed, yielding 1–2 follicles per size class. Consequently, a total of ten follicles were collected for each size class, derived from at least five different individuals. For single-follicle matched sampling, each follicle was transferred individually to a fresh droplet of 0.1% PVA-PBS. The follicular wall was longitudinally opened using 120 mm fine sharp-tipped forceps, and the innermost intact MGC layer was gently peeled away. Crucially, to account for the nested structure of the data and prevent pseudoreplication, the oocyte, MGCs, and CCs isolated from each individual follicle were subjected to independent RNA library construction and sequencing. Follicles showing any features of atresia-reduced fluid transparency with flocculent, turbid contents, loosening or fragmentation of the mural granulosa layer, and COCs exhibiting reduced cumulus investment or degenerative morphology were excluded (Fig. S1C). Only morphologically healthy follicles with intact granulosa architecture were retained. From each healthy follicle, three matched samples were collected for RNA-seq: MGCs, CCs and oocyte. First, MGCs were reserved for RNA-seq. The corresponding COCs were removed from the same opened follicle, and CCs were mechanically dissociated from the oocyte using a finely drawn glass needle. Approximately 30 tightly adherent cumulus cells immediately surrounding the oocyte were collected as the CC sample for RNA-seq. The denuded oocyte was individually isolated for RNA-seq. All collected cellular materials were promptly transferred into sterile microtubes and stored at −80 °C until further processing.

### Library preparation and RNA sequencing

For each follicle, a single denuded oocyte and its associated pooled CCs were obtained and processed using the SMART-seq2 protocol separately [22, 23]. Each single oocyte and the corresponding CC pool was transferred into a 5.5-μL lysis/primer mixture (1 μL dNTP Mix, 1 μL Oligo(dT) VN primer, 3.5 μL sample buffer; Single Cell Full-Length mRNA Amplification Kit, Cat. No. N712, Vazyme) and reverse transcription was carried out immediately according to the manufacturer’s protocol. The PCR cycles of single oocyte samples and CC samples were 15 and 12, respectively. Amplified cDNAs were purified with VAHTS DNA Clean Beads (N411, Vazyme). Libraries were constructed from 1 ng purified cDNA using the TruePrep DNA Library Prep Kit V2 for Illumina (TD503, Vazyme). After enzymatic fragmentation and end repair/adaptor ligation (per kit instructions), library fragments were PCR-amplified for 15 cycles. Size selection (300–700 bp) was performed with VAHTS DNA Clean Beads, and library quality was assessed by Qubit 3.0 fluorometry, quantitative real-time PCR, and an Agilent 4200 Bioanalyzer. Freshly isolated MGCs from the same follicles (matched to each oocyte and its associated pooled CCs) were collected separately. Because these samples provided sufficient cell number for standard bulk RNA sequencing, unlike the low-input SMART-seq2 protocol required for oocytes and CCs, MGCs were directly submitted to a service provider (BerryGenomics, Beijing, China) for total RNA extraction and subsequent library construction process. All libraries were sequenced on an Illumina NovaSeq 6000 platform.

### Data preparation and principal component analysis (PCA)

Libraries were sequenced on an Illumina platform to generate raw FASTQ reads, which underwent adapter trimming, low-quality and short-read filtering, and quality assessment to produce the clean paired-end RNA-seq reads. Clean paired-end RNA-seq reads were aligned to the *Bos taurus* reference genome (ARS-UCD1.2) using STAR (v2.7.11b) with default settings except that only uniquely mapping reads were retained (--outFilterMultimapNmax 1). Compressed FASTQ files were streamed with --readFilesCommand zcat, and alignments were produced as coordinate-sorted BAM files (--outSAMtype BAM SortedByCoordinate) [24]. Non-strand-specific wiggle coverage files were also generated for visualization. Gene-level read quantification was performed with featureCounts (Subread) using parameters -t exon -g gene_id against the corresponding GTF annotation [25]. The count matrix was analyzed with a Seurat based workflow (v4.4.0) using R (v4.3.2). Cells meeting any of the following criteria were deemed low quality and removed (Fig. S1D): total UMI/read count (nCount_RNA) < 20,000; detected genes (nFeature_RNA) < 1,000; mitochondrial transcript proportion (percent.mt) > 30%. Based on mapping rates and mitochondrial sequence percentages, 3 follicles were excluded across different sample types. A comprehensive summary matrix detailing our sample collection, quality control, and final inclusion numbers is provided in Table S1. Next, the top 2,000 highly variable genes were selected with FindVariableFeatures function [26]. PCA based on the top 2,000 genes was performed to visualize differences among MGCs, CCs, and oocytes using OmicShare Tools [27] (http://www.omicshare.com/tools), an online data analysis platform.

### Identification of differentially expressed genes (DEGs)

Differential expression analysis was conducted using DESeq2 on raw count data. Genes with |fold change| > 2 and an FDR-adjusted *P* value < 0.05 were defined as DEGs. For expression quantification and filtering, gene expression abundance was estimated using transcripts per million (TPM) normalization. Genes were retained for downstream analysis if they exhibited TPM > 1 in at least 15 samples in any one of the three cell types (MGCs, CCs, or oocytes).

### Noise-resistant soft clustering

Noise-robust fuzzy c-means clustering (Mfuzz v2.8.0) was applied to MGCs, CCs, and oocytes from follicles in three size classes (3–5 mm, 6–8 mm, 9–12 mm), yielding eight or six gene expression clusters. Data visualization was performed using R package "ClusterGVis".

### Genes functional enrichment analysis

DEGs and Mfuzz defined stage specific genes were analyzed for Gene Ontology (GO) Biological Process and Kyoto Encyclopedia of Genes and Genomes (KEGG) over representation using a hypergeometric test (Benjamini–Hochberg nominal *P* < 0.05). Gene Set Enrichment Analysis (GSEA, genes ranked by decreasing log_2_FC) identified pathways with |NES| > 1, nominal *P* value < 0.05, and FDR *q* < 0.25. GO term enrichment analysis, KEGG pathway enrichment analysis, KEGG pathway network enrichment analysis and GSEA were performed using Omicshare platform tools [27] (https://www.omicshare.com/tools).

### Ligand-receptor interaction analysis

A catalogue of approximately 1,800 unique *Bos taurus* ligand-receptor interactions was compiled by integrating ligand-receptor pairs from the IUPHAR and FANTOM5 databases, supplemented with additional pairs identified through manual literature curation. Depending on the analytical objective, two related but distinct threshold strategies were used. First, to compare the communication-related transcriptional repertoire among MGCs, CCs, and oocytes, a ligand or receptor gene was considered expressed when its TPM was ≥ 1. This threshold was used only to summarize the number of expressed ligand and receptor genes in each cell type and developmental stage. To evaluate the robustness of this criterion, we repeated the analysis using both relaxed and stringent expression thresholds, which yielded consistent overall patterns. Second, for cell–cell interaction inference, ligand-receptor pairs were scored without applying a strict TPM ≥ 1 pre-filter to each individual ligand or receptor. This strategy was chosen to avoid excluding biologically important interactions involving lowly expressed ligands or receptors. For each ordered ligand-receptor pair, the interaction score was calculated as the product of ligand TPM in the source cell and receptor TPM in the target cell. Ligand-receptor pairs with an interaction score > 1 were retained as putative interactions. Sensitivity analyses using relaxed and stringent interaction-score thresholds showed that the major cell–cell communication patterns were preserved across thresholds. Chord diagrams were used to depict, for each of the three follicular stages, the relative strengths of autocrine and paracrine signaling among MGCs, CCs, and oocytes based on the interaction scores. For any specified interaction direction (e.g., MGC autocrine), overlaps of ligand-receptor pairs across the three follicular stages were shown with Venn diagrams plotted by an online platform for data analysis and visualization (https://www.bioinformatics.com.cn), whereas within a given follicular stage the overlaps among multiple directional interaction sets were summarized using UpSet plots produced by the OmicShare platform. Heatmaps generated in R were employed to display the interaction strengths of selected receptor-ligand pairs either across different follicular stages or between different cell-type pairs. Heterogeneity of ligand-receptor interactions among individual follicles was assessed by recomputing presence at the single-follicle level and displaying overlaps with Venn diagrams.

### Ligand-receptor-pathway analysis

Ligand-receptor-pathway association analysis was performed to infer potential downstream transcriptional programs associated with stage-enriched intercellular interactions. First, ligand-receptor interaction scores across small, medium, and large follicles were visualized as heatmaps and grouped by hierarchical clustering. Based on the stage-dependent clustering patterns, ligand-receptor pairs showing relatively higher interaction scores at a specific follicle stage were classified as small follicle-high, medium follicle-high, or large follicle-high interaction groups. In parallel, stage-specific highly expressed genes in each cell type were identified using MFUZZ clustering and subjected to KEGG pathway enrichment analysis. For each communication direction, receptors from a given stage-enriched ligand-receptor group were compared with the genes annotated to KEGG pathways enriched among the corresponding stage-specific highly expressed genes in the signal-receiving cell. A receptor-pathway link was established only when the receptor gene was included among the genes contributing to an enriched KEGG pathway. For example, for small follicle-high MGC-to-oocyte interactions, receptors from small follicle-high MGC-to-oocyte ligand-receptor pairs were compared with the genes annotated to KEGG pathways enriched among small follicle-high genes in oocytes. If a receptor gene was present in one of these enriched KEGG pathways, the receptor was connected to that pathway. The same procedure was applied to medium follicle-high and large follicle-high interactions, as well as to all other communication directions using the corresponding signal-receiving cell type. The resulting ligand-receptor-pathway associations should be interpreted as inferred links between stage-enriched ligand-receptor interactions and stage-specific transcriptional programs in the receiving cells, rather than direct evidence that a specific ligand-receptor pair activates a given pathway. Ligand-receptor-pathway cascades were visualized with Sankey diagrams (https://www.bioinformatics.com.cn) to illustrate hierarchical relationships.

### MGC culture and cell viability assay for functional evaluation of candidate factors

To evaluate the biological relevance of the transcriptome-inferred ligand-receptor interaction network, versican (VCAN) and intercellular adhesion molecule 4 (ICAM4) were selected for functional validation based on their high expression and predicted roles in MGC autocrine and oocyte-to-MGC communication. MGCs were isolated from 2–8 mm follicles (corresponding to the small and medium stages analyzed in our main functional results) by aspirating follicular fluid with a sterile 20-mL syringe (18‑gauge needle) into 50-mL tubes maintained at 37 °C, where COCs were rapidly identified and hand‑picked under a stereomicroscope, and the residual fluid was passed through a 70-µm strainer. The filtrate was centrifuged at 500 × *g* for 5 min to yield a cell pellet that was subjected to erythrocyte lysis (1 mL expanded to 5 mL total), recentrifuged, washed once with DPBS containing 1% (v/v) penicillin–streptomycin, and resuspended in DMEM/F12 supplemented with 5% (v/v) FBS, 1% (v/v) penicillin–streptomycin, and 1% (v/v) Primocin. Viable cells were enumerated by trypan blue exclusion and seeded into 60‑mm dishes at 1 × 10^5^–1 × 10^6^ cells/mL, cultures were maintained at 37 °C in a humidified 5% CO_2_ atmosphere.

Adherent MGCs were detached, counted, and reseeded into flat‑bottom 96‑well plates at 5 × 10^3^ viable cells per well (100 µL/well) and cultured in growth medium containing either recombinant VCAN (R&D Systems, Minneapolis, MN, USA) or recombinant ICAM4 (R&D Systems, Minneapolis, MN, USA) at final concentrations of 0, 10, 50, 100, 500, or 1,000 ng/mL. Cell viability assays were performed using a CCK-8 assay (Beyotime, Biotechnology, Shanghai, China) [28]. After incubation for 48 h, 10 µL of CCK-8 solution was added to each well, and the cells were incubated for another 2 h. The cell viability was calculated in an enzyme-linked immunosorbent assay reader (Infinite F200, TECAN, Männedorf, Switzerland) done in triplicate by measuring the OD_450_.

### In vitro maturation, fertilization and culture

COCs were isolated from 2–8 mm follicles by aspirating follicular fluid with a sterile 20-mL syringe (18‑gauge needle). Only morphologically intact COCs were randomly distributed among the different IVM treatment groups prior to culture. Routine IVM was performed as previously described [29]. Briefly, COCs were in vitro matured in TCM199 supplemented with 0.02 IU/mL porcine FSH (Folltropin-V; Bioniche Animal Health, Belleville, Ontario, Canada), 0.02 IU/mL LH (Sigma-Aldrich, St Louis, MO, USA), 1 μg/mL E_2_ (Sigma-Aldrich, St Louis, MO, USA) and 10% (v/v) FBS for 24 h at 38.8 °C in a humidified atmosphere with 6% CO_2_ in air (at least 15 COCs per 100 μL drop). PTN and jagged canonical notch ligand 1 (JAG1) were selected as candidate factors from the predicted intercellular communication network and were supplemented during bovine IVM to evaluate their potential effects on oocyte maturation and subsequent embryonic development. The JAG1 concentration range was designed based on the typical working concentration for most recombinant proteins in vitro (around 100 ng/mL), whereas the PTN range was based on our preliminary dose-exploration experiments. Oocytes were subjected to IVM in a basic culture medium supplemented with varying concentrations of recombinant PTN (0, 50, 100, or 200 ng/mL; R&D Systems, Minneapolis, MN, USA) and JAG1 (0, 1, 10, 100, or 200 ng/mL; R&D Systems, Minneapolis, MN, USA). After maturation, the COCs were washed three times with BO (Bracket and Oliphant) washing medium and transferred to BO fertilization medium consisting of 20 μg/mL heparin sodium and 6 mg/mL fatty acid-free BSA. Frozen semen was thawed at 37 °C and washed twice by centrifugation at 320 × *g* for 5 min. After the final wash, the motility and concentration of the sperm were determined and added to each fertilization drop, achieving a total concentration of 2 × 10^6^ spermatozoa/mL. The oocytes and sperm were incubated together for 8 h at 38.8 °C in a humidified atmosphere with 6% CO_2_ in air. In each biological replicate of the in vitro fertilization experiment, three straws of frozen semen from at least two bulls were mixed before insemination to minimize bull-to-bull variation in fertilization capacity and subsequent embryo development [30]. After fertilization, the presumptive zygotes were transferred to 100 μL mSOFaa culture medium droplets containing 5 mg/mL fatty acid-free BSA for 2 d and subsequently supplemented with 10% (v/v) FBS from day 3 until day 8 at 38.8 °C in a humidified atmosphere of 6% CO_2_ (v/v), 6% O_2_ (v/v), and 88% N_2_ (v/v) [31]. The percentage cleavage and rates of subsequent embryo development to the blastocyst stage were recorded on day 2 and 8, respectively. For all IVM and embryo development assays, experiments were performed in at least three independent biological replicates, with a minimum of 45 oocytes per group per replicate.

### Immunofluorescence staining for the quantification of blastocyst cells

Collected blastocysts were washed three times with 0.1% PVA-PBS and then washed with acidic Tyrode’s solution to eliminate the zona pellucida. Then blastocysts were fixed in 4% paraformaldehyde in PBS overnight at 4 °C. After being permeabilized with 0.5% Triton X-100 in 0.1% PVA-PBS, blastocysts were blocked in 1% BSA in 0.1% PVA-PBS for 1 h, then sequentially incubated with primary antibody overnight at 4 °C. Next, the blastocysts were washed three times with 0.1% PVA-PBS for 5 min and incubated with secondary antibodies for 1 h at room temperature. Finally, the samples were treated with DAPI for 5 min, mounted with coverslips. Images were recorded using a fluorescence microscope (BX51TRF; Olympus, Tokyo, Japan). Anti-CDX2 (1:500 dilution, BioGenex-MU392A-5UC) and anti-SOX2 (1:500 dilution, eBioscience-14-9811-82, Thermo Fisher Scientific) were used as primary antibodies.

### Fluorescence staining and quantitative analysis of transzonal projections (TZPs)

To evaluate the effect of PTN on TZP density, COCs were cultured in IVM medium with or without 100 ng/mL PTN for 6 h. After collection, the cumulus cells were removed, and the oocytes were washed three times in 0.1% PVA-PBS. The oocytes were then fixed in 4% paraformaldehyde (PFA) for 30 min at room temperature. Following fixation, samples were washed with 0.1% PVA-PBS and permeabilized in PBS containing 0.5% Triton X-100 for 30 min at room temperature. After permeabilization, the oocytes were transferred to a working solution of Rhodamine-Phalloidin and incubated in the dark for 2 h at room temperature to fully label the F-actin microfilaments constituting the TZPs. After staining, the samples were thoroughly washed three times with 0.1% PVA-PBS to remove unbound background dye. The oocytes were then placed on glass slides, slightly compressed, and mounted with coverslips. Images were acquired using a laser scanning confocal microscope, focusing specifically on the slender actin filaments penetrating the zona pellucida.

### Quantification and statistical analysis

All fluorescence signals and image analyses were quantified using ImageJ software (Rawak Software Inc., Stuttgart, Germany). Data are presented as the mean ± SEM. For comparisons between two independent groups, an unpaired Student's *t*-test was utilized. For comparisons involving three or more experimental groups, an ANOVA followed by Tukey’s post hoc test was applied to evaluate statistical significance. Differences were considered statistically significant when *P* < 0.05. Detailed information regarding the number of biological replicates, sample sizes, and specific statistical tests for each experiment is provided in the corresponding figure legends.

## Results

### The mural granulosa cells are highly dynamic and oocytes are relatively stable during bovine antral follicle development

**Fig. 1:**
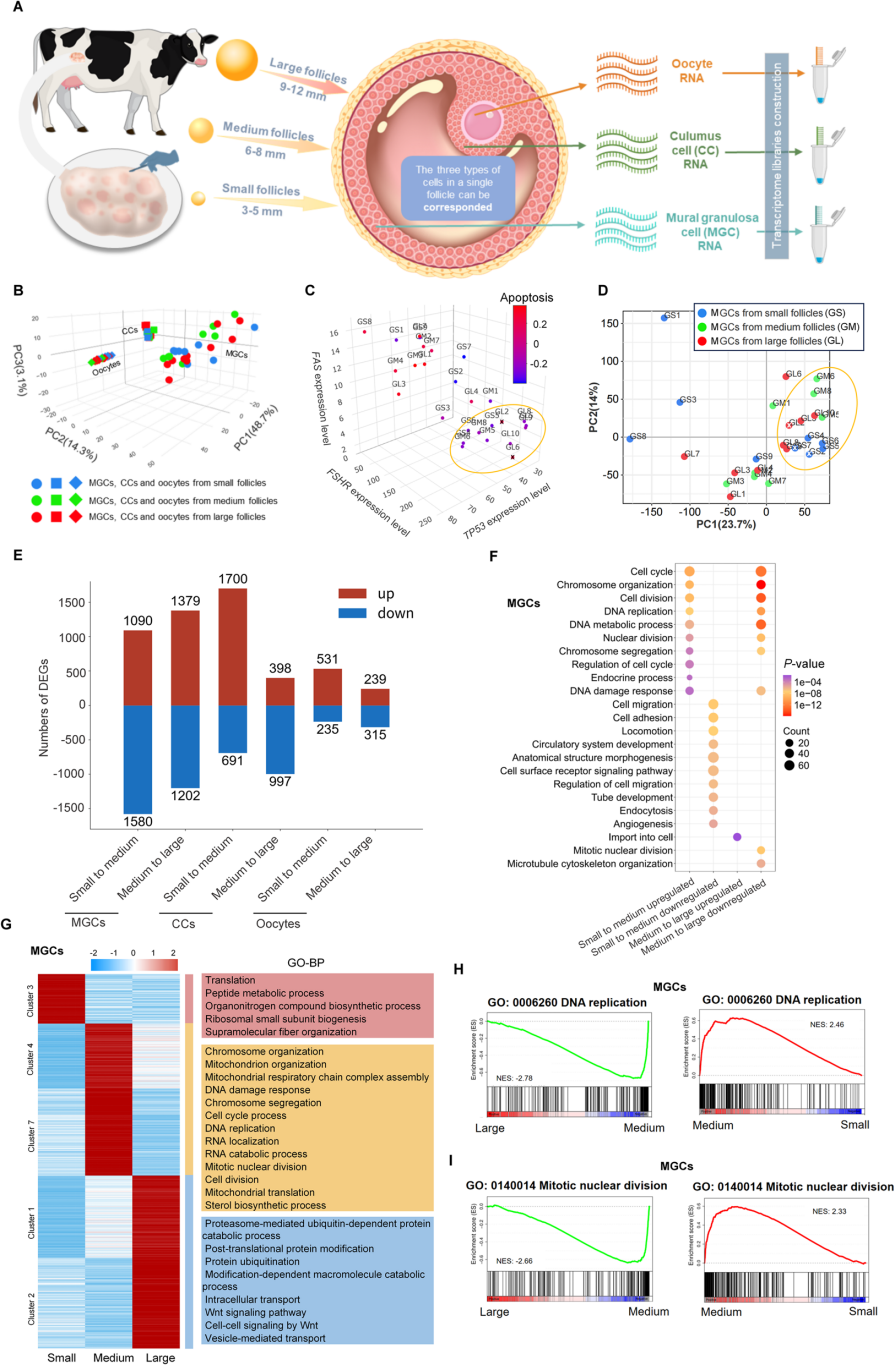
The global transcriptome patterns and gene expression dynamics of bovine MGCs, CCs and oocytes. **A** Schematic illustration showing the preparation of bovine MGCs, CCs and oocytes prepared from follicles of different diameters (3–5 mm, 6–8 mm, 9–12 mm) for RNA-seq. **B** Principal component analysis (PCA) of RNA-seq data of bovine MGCs, CCs and oocytes prepared from follicles of different diameters. Points are colored by follicle diameter group and shaped by cell type (see legend). The 3D PCA (PC1–PC3) clearly separates MGCs, CCs, and oocytes. Axis labels indicate the percentage of variance explained by each principal component. **C** 3D scatter plot of bovine mural granulosa cells (MGCs) positioned by gene expression: *FSHR*, *TP53*, and *FAS* expression level. Each follicle contributes one point (its MGCs), with point color encoding apoptosis activity (see legend). A yellow outline highlights follicles whose MGCs show high FSHR and low TP53/FAS expression; two points with elevated apoptosis within this group were excluded from the highlight. Labels denote sample identity: G: MGCs; GS3: MGCs from the 3^rd^ small follicle; GM5: 5^th^ medium follicle; GL8: 8^th^ large follicle. **D** PCA (2D) of RNA-seq data of bovine MGCs prepared from follicles of different diameters. **E** Numbers of differentially expressed genes (DEGs) across follicular growth (small → medium → large) in MGCs, CCs, and oocytes. DEGs were defined as |Fold Change| > 2 with *P* value < 0.05. **F** GO biological process (BP) enrichment of DEGs in MGCs across follicular growth (small → medium → large). **G** Heatmap of stage-specific high-expression genes in MGCs and corresponding GO-BP enrichment. **H **and** I** GSEA of dynamic changes in DNA replication (**H**) and mitotic nuclear division (**I**) in MGCs across follicular development (small → medium → large). The curves show the enrichment profiles of the indicated NESs in each panel, with nominal *P* value close to 0

To investigate the dynamic intrafollicular dialogue across different somatic cellular compartments and oocytes, we collected bovine antral follicles at sequential stages. Following a strict two-step morphological screening prior to sequencing to ensure gross follicular health, we dissected oocytes, MGCs and CCs from individual follicles (Fig. 1A and Fig. S1A, B). Then, RNA-seq was performed, and the PCA clearly separated oocytes, MGCs and CCs (Fig. 1B), in line with their cell type-specific expression of the well-known markers (Fig. S1E). To ensure that subsequent analyses were restricted to healthy follicles, we applied both marker-based filtering and transcriptome-level validation. Follicles containing MGCs with reduced expression of FSHR and elevated expression of TP53 and FAS were classified as subordinate follicles entering the early molecular stages of atresia. Consequently, the entire matched set of cells (MGCs, CCs, and the oocyte) from these specific follicles was excluded from the main developmental analyses, as these features are associated with compromised follicular health (Fig. 1C, D and Fig. S1F, G, I) [28, 29]. Given that estrogen production capacity—primarily driven by CYP19A1 (aromatase) in MGCs—is a key hallmark of dominant, healthy follicles, we further performed correlation analyses using CYP19A1 expression and steroidogenic pathway activity as transcriptomic proxies of follicular function. Consistent with this framework, CYP19A1 expression showed a significant positive correlation with FSHR across follicles, and a strong negative correlation with the atresia-associated markers TP53 and FAS. Moreover, at the pathway level, the enrichment score for "steroid hormone biosynthesis" was significantly negatively correlated with the "apoptosis" pathway in MGCs. Together, these results provide multi-level evidence that the follicles included in our study represent a physiologically healthy population, supporting the validity of downstream analyses.

Dynamic comparisons of consecutive stages for oocytes, MGCs, and CCs showed that MGCs underwent continuous and dramatic changes, as revealed by the large number of upregulated and downregulated DEGs, while oocytes maintained a relatively stable state in comparisons to MGCs and CCs (Fig. 1E). This pattern was also reflected in pseudotime analysis (Fig. S1H), in which MGC samples showed clearer stage-associated separation. These results suggest that MGCs exhibit the most extensive transcriptomic shifts and possess the largest repertoire of dynamic genes during follicle development.

When focusing on DEGs in MGCs, we found a stage-specific increase in cell cycle and associated processes during the transition from small to medium follicles, followed by a sharp decline thereafter (Fig. 1F). Next, we investigated the dynamic gene expression patterns of oocytes, MGCs, and CCs across three developmental stages by using non-hierarchical Mfuzz clustering analysis (Fig. S2A), and analyzed functions of genes that were preferentially expressed at specific developmental stages using Gene Ontology (GO). In line with the aforementioned dynamic comparisons (Fig. 1E), MGCs in medium follicles showed higher proliferation because the most significant GO terms in clusters 4 and 7 included cell cycle processes, DNA replication, mitotic nuclear division, etc. (Fig. 1G–I). In CCs, in addition to translation and relevant processes, such as ribonucleoprotein complex biogenesis, a noteworthy change was the energy shift from mitochondrial respiration to glycolysis (i.e., enrichments of mitochondrial functions and glycolysis in small and large follicles, respectively) (Fig. S2B). We further evaluated hypoxia-associated signatures at the transcriptomic level. GSVA indicated an increase in HIF-1α signaling activity in CCs from large follicles (Fig. S2D), and GSEA showed enrichment of processes related to "response to hypoxia" and "cellular response to hypoxia" (Fig. S2E, F). In oocytes, RNA processing and translation, as well as protein processing and localization, were the most significant GO terms in small and medium follicles, while chromatin remodeling and associated terms are more enriched in large follicles (Fig. S2C).

### Bovine antral follicles exhibit spatiotemporal commonality in intercellular communications

**Fig. 2:**
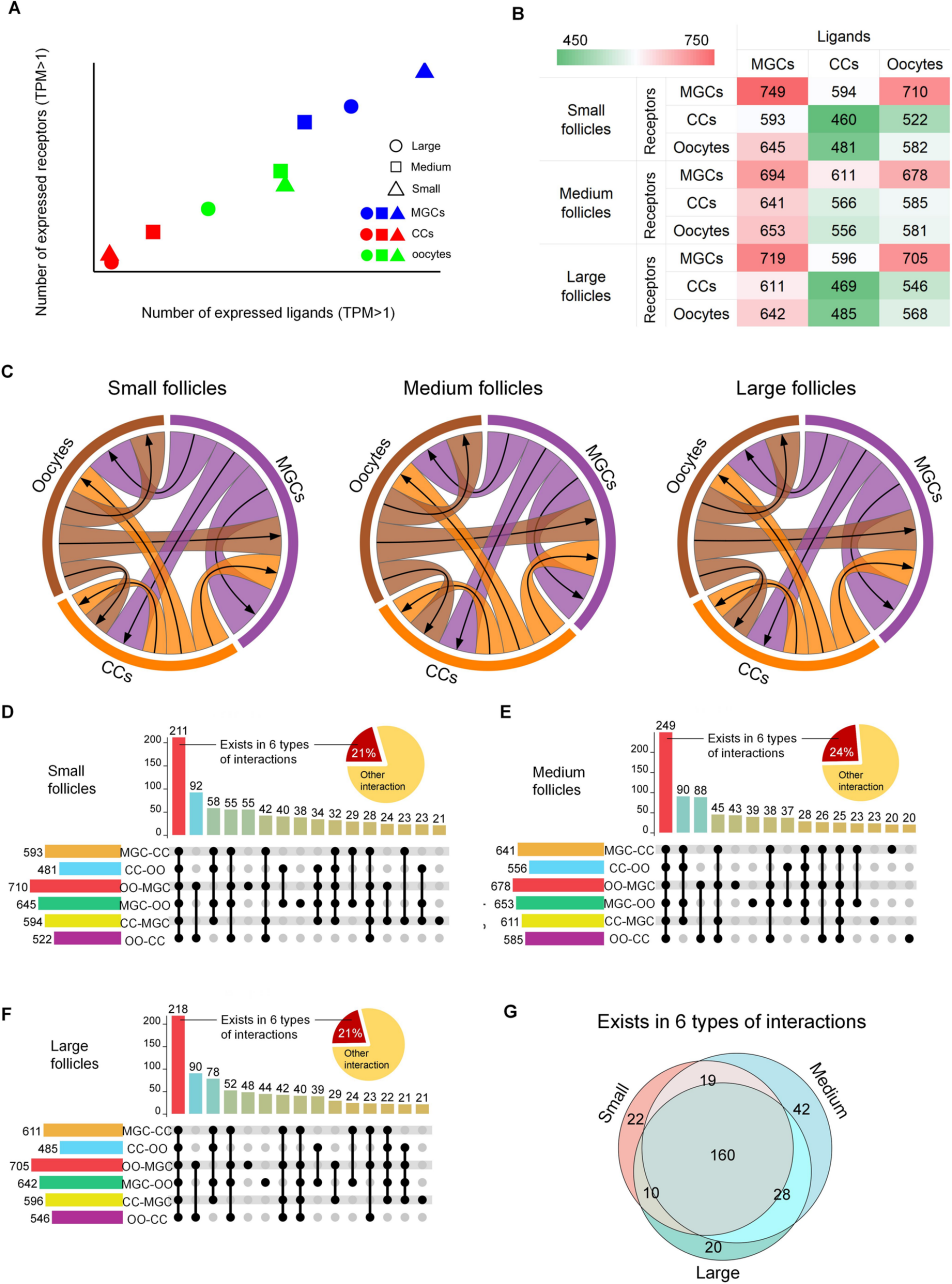
Bovine antral follicles exhibit spatiotemporal commonality in intercellular communications. **A** Scatter plot showing the number of ligands and receptors as coordinates for three types of cells (MGCs, CCs, and oocytes) across three stages of follicular development. **B** Heatmap showing the number of ligand-receptor pairs between three types of cells (MGCs, CCs, and oocytes) across three stages of follicular development. Ligand-receptor pairs with an interaction score (ligand expression × receptor expression) greater than 1 are included, with the color gradient representing the quantity of these interactions. **C** Chord diagrams depicting the interaction relationships among three types of cells at three stages of follicular development. Sector size indicates the total number of predicted interactions involving each cell type, and chord width indicates the number of predicted ligand-receptor pairs between the connected cell types. The arrow in the chord indicates the orientation of ligands from sender cells toward receptors on receiver cells. **D–F** Upset plots showing the distribution of ligand-receptor pairs across six interaction types (between MGCs, CCs, and oocytes) at three stages of follicular development: small (**D**), medium (**E**), and large (**F**) follicles. **G** Venn diagram illustrating the overlap of ligand-receptor pairs present in all six interaction types across three stages of follicular development: small, medium, and large follicles

To elucidate the intrafollicular crosstalk among oocytes, MGCs, and CCs, we used a well-established approach that characterizes intercellular communication by profiling and scoring ligand-receptor interactions across cell types [32, 33]. Quantitation of the number of expressed ligands and receptors demonstrates that MGCs exhibited the highest number of detected ligand-receptor transcripts across antral follicle development, followed by oocytes, whereas CCs showed comparatively fewer (Fig. 2A). In this study, the number of expressed ligand and receptor genes in each cell type at different stages was used as an integrated measure to reflect their relative involvement in intercellular communication during follicular development. Based on this criterion, MGCs displayed a broader communication-related transcriptional repertoire compared to other cell types. To ensure the robustness of this analysis, both relaxed and stringent thresholds were applied to define expressed genes, and consistent patterns were observed across these criteria (Fig. S3A, B).

We next constructed an interaction matrix by calculating predicted autocrine and paracrine interactions between each two cell types, including the autocrine pattern. MGCs exhibited the highest number of interaction pairs, particularly in autocrine interactions, as well as in interactions where MGCs act as secreting cells and oocytes or CCs as receiving cells (Fig. 2B, C and Fig. S3C, D). Another interesting finding is that oocytes also contributed substantially to ligand expression in oocyte-to-MGC interaction pairs, suggesting their potential involvement in creating the unique follicular microenvironment.

Intriguingly, intrafollicular communications showed high commonality upon focusing on diverse intercellular communications at each stage. We quantified the number of ligand-receptor pairs involved in distinct paracrine communication modes. The Upset plot showed that a great proportion of ligand-receptor pairs were shared in all six paracrine interaction modes at each developmental stage (Fig. 2D–F). By contrast, pairs restricted to single or dual interaction modes contributed a small proportion. In line with this, Venn analysis of expressed genes in the three cell types at each stage indicates that over 55% of total expressed genes (small: 58.34%; medium: 58.68%; large: 64.23%) were co-expressed in all three cell types (Fig. S3E–G). These results suggest that antral follicles employ a spatial commonality strategy across different communications among cell types. Furthermore, intersection analysis of the intrafollicular common ligand-receptor pairs across all three stages demonstrates that more than 50% (160/301) of shared pairs are stably present throughout antral follicle development, thus revealing both temporal and spatial commonality (Fig. 2G and Fig. S4).

### Constructed map of intrafollicular ligand-receptor-pathway cascades shows many-to-many interaction patterns

**Fig. 3:**
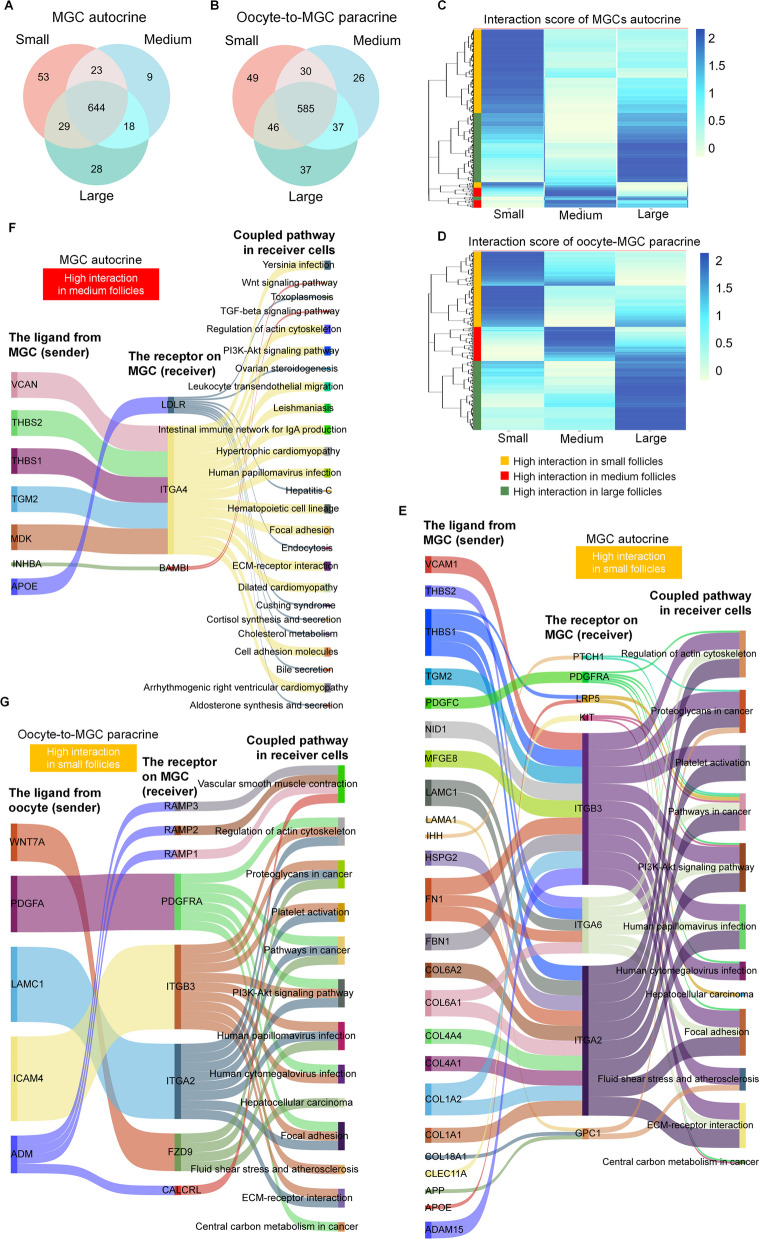
Constructed map of intrafollicular ligand-receptor pathway cascades shows a redundant interaction pattern. **A** Venn diagram showing the overlap of ligand-receptor pairs involved in MGC autocrine interactions across three stages of follicular development: small, medium, and large follicles. **B** Venn diagram illustrating the overlap of ligand-receptor pairs involved in Oocyte-to-MGC paracrine interactions across three stages of follicular development: small, medium, and large follicles. **C** Clustered heatmap showing the interaction scores of ligand-receptor pairs involved in MGC autocrine interactions. **D** Clustered heatmap showing the interaction scores of ligand-receptor pairs involved in oocyte-to-MGC paracrine interactions. **E** Sankey diagram illustrating the ligand-receptor-pathway relationships in MGC autocrine interactions within the small follicle stage. **F** Sankey diagram illustrating the ligand-receptor-pathway relationships in MGC autocrine interactions within the medium follicle stage. **G** Sankey diagram illustrating the ligand-receptor-pathway relationships in oocyte-to-MGC paracrine interactions within the small follicle stage

Focusing on MGC autocrine and oocyte-to-MGC interactions cross stages—the two communication axes exhibiting the highest number of predicted interaction links (Fig. 2B), we found the majority of interactions, i.e., 80.10% of MGC-autocrine pairs and 72.22% of oocyte-to-MGC interactions remained present throughout antral follicle development (Fig. 3A, B). Despite their spatiotemporal commonality, the high-scoring interaction pairs displayed notable stage-specific dynamics (Fig. 3C, D). We next constructed a directional map of ligand-receptor interactions and their coupled downstream pathways for MGC autocrine and oocyte-to-MGC communications across small, medium, and large follicles. For MGC-autocrine interactions, the most prominent high-scoring interacting pairs involved integrin (ITG)-family receptors, which were computationally associated with numerous downstream pathways and processes that have been reported to be important for ovarian folliculogenesis, such as cytoskeleton regulation, PI3K-Akt signaling, cell adhesion and cholesterol metabolism (Fig. 3E, F and Fig. S5A) [34, 35]. It is particularly noteworthy that ITG receptors in MGC autocrine interactions were predicted to be associated with multiple ligands, suggesting that ITG-mediated signaling may represent a prominent and extensively connected component of the inferred interaction network in MGCs. For example, our transcriptomic data predicted that ITG4 in medium follicular MGCs could be associated with multiple autocrine ligands, including Versican (VCAN), Thrombospondin 1/2 (THBS1/2), Transglutaminase 2 (TGM2), Midkine (MDK), thus potentially linking to a great deal of downstream intracellular pathways (Fig. 3F), while THBS1 in small follicular MGCs was predicted to interact with multiple receptors ITGB3, ITGA6 and ITGA2, which were bioinformatically associated with many fundamental cellular processes (Fig. 3E).

For oocyte-to-MGC interactions, many ligand-receptor pairs also showed many-to-many interaction pattern. For example, oocyte-secreted ADM was inferred to interact with many receptors such as RAMP1, RAMP2, RAMP3 and CALCRL, thus potentially participating in the modulation of MGC cytoskeleton in small follicles (Fig. 3G); In large follicles, NODAL and INHA were predicted to regulate fluid shear stress and TGF-beta signaling via the same interacting receptor ACVR2A (Fig. S5B).

Intriguingly, besides the basal processes such as cytoskeleton regulation, adhesion junction, carbon metabolism, cell cycle and proliferation (enriched in cancer-related terms), we unexpectedly noticed that oocytes might play a unique role in immune regulation of MGCs. Although it has been reported that MGCs have innate immune capabilities, which contribute to their role in folliculogenesis [36, 37], the underlying mechanism remains poorly understood. Our transcriptomic results raise the possibility that oocyte-derived ligands may be linked to immune-related transcriptional programs in MGCs through putative VWF/ICAM4-ITGA2B and HBEGF-EGFR interactions. (Fig. S5B).

Finally, to assess whether the transcriptome-derived interaction map could inform biologically relevant signaling candidates, we selected VCAN and ICAM4, two highly expressed ligands predicted to participate in MGC autocrine (Fig. 3F and Fig. S5A, D) or oocyte-to-MGC interactions (Fig. 3G and Fig. S5B, E) for functional evaluation. Given that MGC proliferation is a hallmark event during antral follicular growth, we examined whether these candidate ligands could influence this process. Exogenous supplementation with either VCAN or ICAM4 significantly enhanced proliferation of cultured MGCs, demonstrating that VCAN and ICAM4 may contribute in regulating MGC behavior (Fig. S5F, G). These results provide preliminary functional support that VCAN and ICAM4 can influence MGC proliferation in vitro and may represent biologically relevant candidates from the inferred ligand-receptor interaction network.

### The map of MGC-secreted ligand-receptor pathway cascades shows the similar many-to-many interaction pattern

**Fig. 4:**
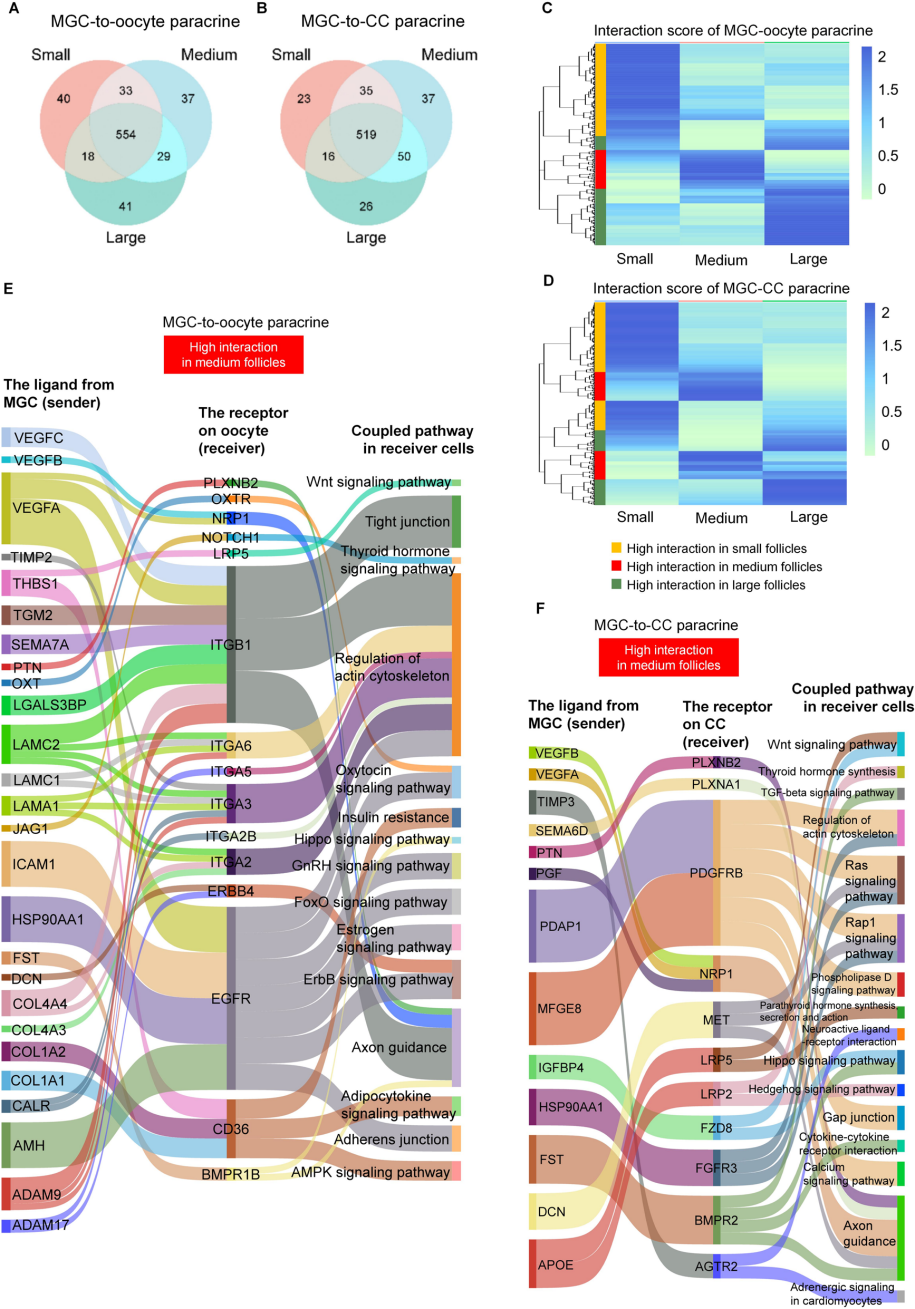
The map of MGC-secreted ligand-receptor pathway cascades shows the similar redundant interaction pattern and their roles in enhancing oocyte developmental competence. **A **and** B** Venn diagrams showing the overlap of ligand-receptor pairs involved in MGC-to-oocyte (**A**) and MGC-to-CC (**B**) paracrine interactions across three stages of follicular development: small, medium, and large follicles. **C **and **D** Clustered heatmaps showing the interaction scores of ligand-receptor pairs involved in MGC-to-oocyte (**C**) and MGC-to-CC (**D**) paracrine interactions. **E **and** F** Simplified representations of the full Sankey diagrams shown in Fig. S6B and Fig. S6E, respectively, focusing on paracrine interactions with high scores in medium follicles. **E** MGC-to-oocyte paracrine ligand-receptor-pathway network. **F** MGC-to-CC paracrine ligand-receptor-pathway network. Disease-related pathways were excluded from these simplified diagrams to enhance readability; no additional filtering was applied to the ligand-receptor interactions beyond the criteria used in the full analysis

As the active cell type exhibiting the broadest repertoire of communication molecules within the follicular environment (Fig. 2A), MGCs are notably absent in conventional IVM culture systems, thus the absence of MGC-secreted ligands may be a major reason for the poor efficiency of IVM [11, 38]. Paralleling MGC autocrine and oocyte-to-MGC interactions, the majority of MGC-secreted interactions—specifically 73.67% of MGC-to-oocyte and 73.51% of MGC-to-CC interactions—persisted throughout antral follicle development (Fig. 4A, B) and displayed notable stage-specific dynamics (Fig. 4C, D). The predicted interactions mediated by MGC-secreted factors similarly involved ITG-family receptors on CCs and oocytes, potentially modulating essential signaling pathways via interconnected interaction networks (Fig. 4E and Fig. S6A–D). Consistently, integration of oocyte transcriptomic data with publicly available proteomic data confirmed the detectable expression of multiple representative receptors on oocytes, including ITG-family members, growth factor receptors, and other signaling receptors, which were grouped according to their relative transcriptomic abundance (Fig. S7A). Within these networks, extracellular matrix ligands-specifically LAMA1/LAMC1 (laminins) and COL1A1/COL1A2 (collagens)- emerged as prominent predicted interactors for ITG-family receptors (Fig. 4E and Fig. S6A–F). Distinct from the autocrine mechanisms observed in medium-follicle MGCs, these inferred paracrine interactions extended beyond ITG receptors: MGC-derived VCAN was predicted to engage TLR2 on CCs (Fig. 4F), while THBS1 was predicted to target LRP5 (Fig. 4E), and MDK was associated with LRP1/2 and ALK on oocytes (Fig. S6C). In addition to ITG-mediated signaling pathway, these predicted paracrine axes were bioinformatically associated with several key cascades, including LRP5-dependent Wnt signaling pathway (Fig. 4E, F), NOTCH1-mediated thyroid hormone signaling pathway (Fig. 4E), and PDGFRB-associated Ras signaling pathway and gap junction regulation (Fig. 4F). Furthermore, IGF1R signaling was predicted to participate in regulating progesterone-mediated oocyte maturation, oocyte meiosis, as well as the MAPK and HIF-1 signaling pathways (Fig. S6F).

### The constructed map enables the prediction of MGC-secreted factors that can improve the oocyte quality after in vitro maturation

**Fig. 5:**
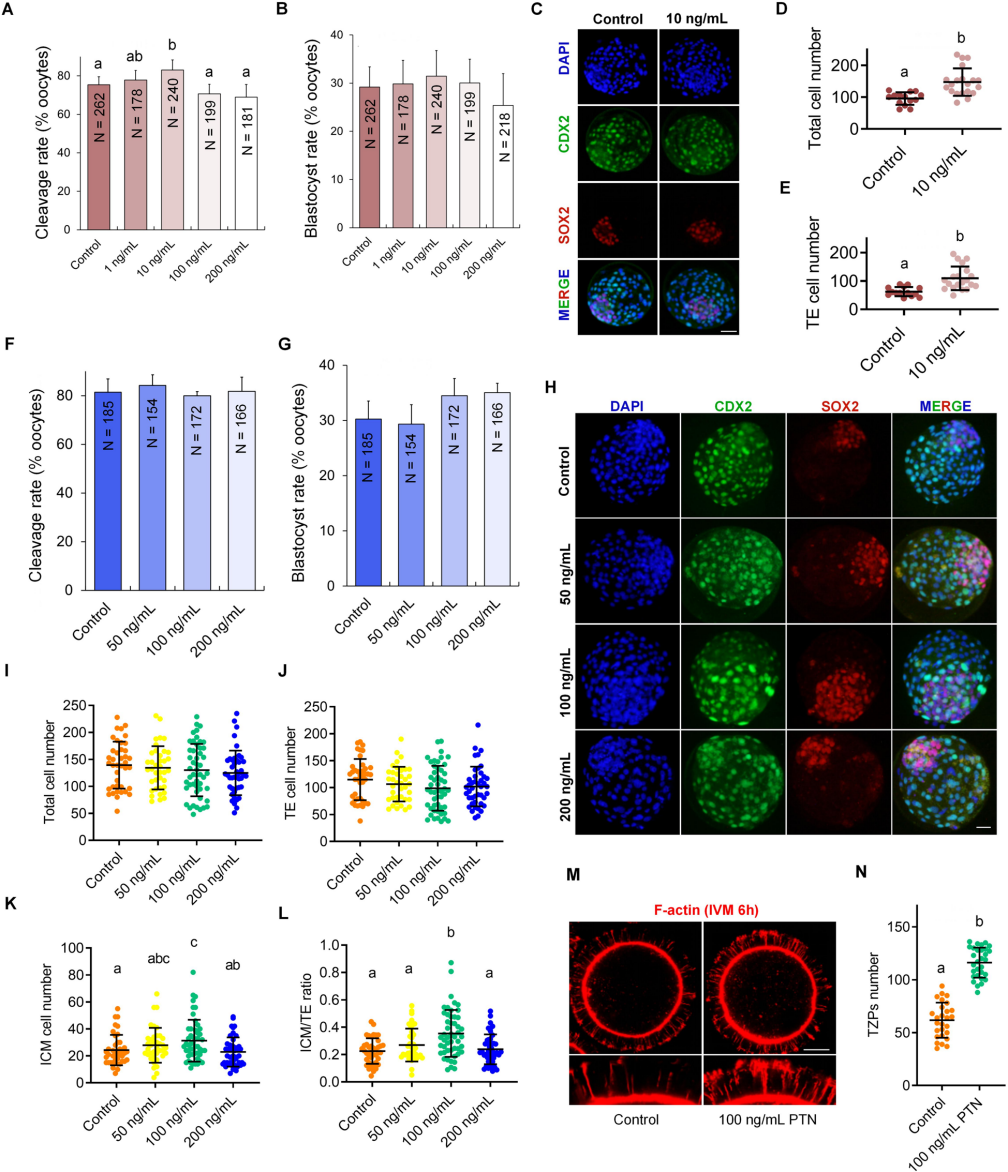
MGC-secreted JAG1 and PTN can improve bovine oocyte IVM quality. **A **and** B** Effect of concentrations of JAG1 in IVM medium ranging from 0 to 200 ng/mL on the percentage of oocytes that cleaved after fertilization (**A**) and percentage of oocytes that became a blastocyst (**B**). **C** Representative immunofluorescence images of blastocysts derived from oocytes matured in IVM medium supplemented with different concentrations of JAG1 (0, 10 ng/mL). DAPI (blue) stains total nuclei, CDX2 (green) marks trophectoderm (TE) cells, and SOX2 (red) labels inner cell mass (ICM) cells. Scale bar = 20 μm. **D **and** E** Total blastocyst cell number and TE cell number in blastocysts derived from oocytes matured in IVM medium supplemented with different concentrations of JAG1. **F **and** G** Effect of concentrations of PTN in IVM medium ranging from 0 to 200 ng/mL on the percentage of oocytes that cleaved after fertilization (**F**) and percentage of oocytes that became a blastocyst (**G**). **H** Representative immunofluorescence images of blastocysts derived from oocytes matured in IVM medium supplemented with different concentrations of PTN (0, 50, 100, 200 ng/mL). DAPI (blue) stains total nuclei, CDX2 (green) marks trophectoderm (TE) cells, and SOX2 (red) labels ICM cells. Scale bar = 20 μm. **I–L** Total blastocyst cell number (**I**), TE cell number (**J**), ICM cell number (**K**), and ICM/TE ratio (**L**) in blastocysts derived from oocytes matured in IVM medium supplemented with different concentrations of PTN. **M** Representative fluorescence images of TZPs stained with F-actin (red) in the control and 100 ng/mL PTN-treated groups after 6 h of IVM. The bottom panels display magnified views of the TZPs crossing the zona pellucida. Scale bar = 50 μm. **N** Quantification of the TZP number per oocyte in the control and PTN-treated groups. For all IVM and embryo development assays, experiments were performed in at least three independent biological replicates, with a minimum of 45 oocytes per group per replicate. Statistical analyses were conducted at the replicate level rather than by pooling all oocytes to ensure biological independence. Data are expressed as mean ± SEM. Statistical significance was determined using one-way ANOVA with Tukey’s post hoc test for multiple comparisons and an unpaired Student's *t*-test for two-group comparisons, with distinct letters indicating significant differences (*P* < 0.05)

We hypothesized that ligands secreted by MGCs targeting receptors on CCs or oocytes may enhance oocyte developmental competence and improve maturation quality in comparison with conventional IVM systems. Based on our interaction networks, we identified JAG1 as a prominent MGC-secreted ligand predicted to interact with the NOTCH1 receptor on oocytes within medium-sized follicles (Fig. 4E). To validate its functional effect, exogenous JAG1 was supplemented into the IVM medium, followed by standardized in vitro fertilization and embryo culture. Oocyte quality was subsequently evaluated based on the developmental rate and the quality of the resulting blastocysts (Fig. 5A, B). Our results demonstrated that supplementation with 10 ng/mL JAG1 led to a significant improvement in the cleavage rate (Fig. 5A). Although the blastocyst rate was not significantly improved (Fig. 5B), the quality of blastocysts—as indicated by total cell number and trophectoderm (TE) cell number—was significantly enhanced by the supplementation of JAG1 (Fig. 5C–E).

Given that improving the blastocyst yield remains a main objective in in vitro embryo production practice, it is necessary to investigate other MGC-secreted ligands within the identified redundant interaction networks (Fig. 4E, F and Fig. S6A–F). While several factors—including VEGFA [39], DCN [40], APOE [41], FST [42], FN1 [43], and MDK [44]—have been previously reported to regulate oocyte maturation through signal transduction, extracellular matrix remodeling, or lipid metabolism. Building on this context, we focused on pleiotrophin (PTN), which concurrently activates the plexin B2 (PLXNB2) receptor—a key component of the axon guidance pathway—on both CCs and oocytes (Fig. 4E, F). The expression pattern of PTN is distinct from that of JAG1, which exhibits peak expression in oocytes (Fig. S6G). Specifically, PTN is predominantly secreted by MGCs, while its expression remains markedly lower in CCs and is almost absent in oocytes (Fig. S6H). Among the 160 interaction pairs that showed both temporal and spatial commonality (Fig. 2G), the PTN-PLXNB2 emerged as particularly noteworthy (Fig. S4). Intriguingly, MDK—a structural homolog of PTN [45]—similarly constitutes a degenerate interaction pair via the MDK-ITGA6/ITGB1 axis. Furthermore, both MGC-secreted PTN and MDK concurrently target the ALK receptor on oocytes within large follicles (Fig. S6C), suggesting a coordinated signaling mechanism during terminal follicular development. Further analysis of PTN-mediated interaction pairs showed prominent PTN-receptor communication across follicular cell types, particularly in MGC-to-oocyte and MGC-to-CC axes in medium follicles (Fig. S7B). Public oocyte proteomic data further showed that PLXNB2 and SDC3 protein abundance was higher at the GV stage than at MII, suggesting that PTN-mediated signaling through these receptors may be more relevant during the GV-stage period of follicular development (Fig. S7C, D).

We hypothesized that exogenous PTN supplementation may enhance oocyte quality after IVM. Then, standardized in vitro fertilization and culture were performed and the developmental rate and quality of blastocysts were used to assess the oocyte quality (Fig. 5F, G). Supplementation with 100 ng/mL PTN showed a tendency to increase the blastocyst formation rate, although this difference did not reach statistical significance (*P* = 0.075; Fig. 5G). In addition, PTN supplementation significantly increased both inner cell mass (ICM) cell number and ICM/TE cell ratio, the critical indicator of blastocyst quality. Given that the ICM is the sole progenitor of the fetus, this elevated ratio serves as a critical indicator of enhanced blastocyst developmental potential, effectively bridging the quality gap between in vitro-produced and in vivo-derived embryos (Fig. 5H–L).

Prompted by our prediction implicating the axon guidance pathway—which classically governs cellular extensions and actin cytoskeleton remodeling—we hypothesized that PTN-PLXNB2 signaling might modulate transzonal projections (TZPs), the critical physical bridges between CCs and oocytes. To test whether this predicted mechanism occurs in vitro, we evaluated the effect of 100 ng/mL PTN supplementation during the first 6 h of IVM. Notably, the PTN-treated group exhibited a significantly higher number of TZPs compared to the control group (Fig. 5M, N). These findings not only indicate that PTN preferentially enhances oocyte maturation quality by strengthening essential somatic-germ cell communication, but also confirm that our constructed interaction map can effectively predict MGC-derived ligands for improving IVM systems.

### Intrafollicular interactions show heterogeneity among follicles

**Fig. 6:**
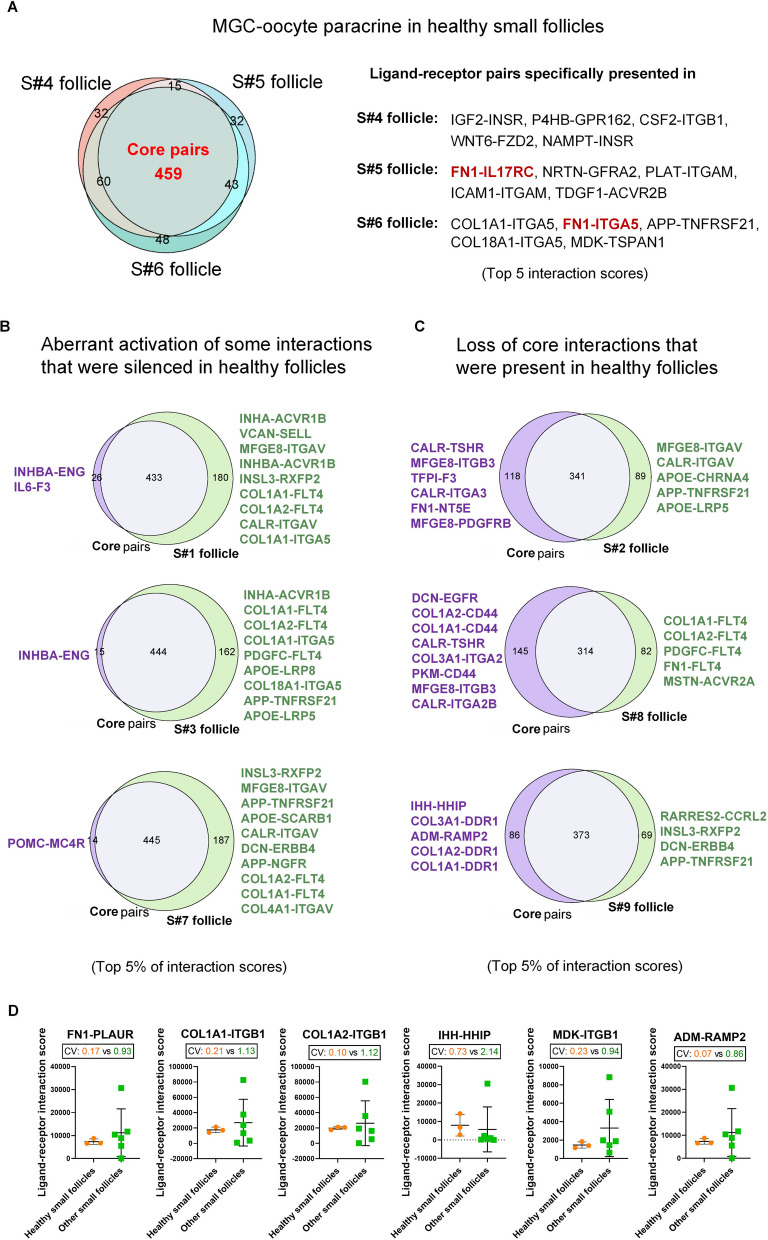
Intrafollicular interactions show heterogeneity among follicles. **A** Venn diagram showing the shared and unique MGC-to-oocyte paracrine ligand-receptor pairs among three healthy small follicles representing this developmental stage. **B** Venn diagram illustrating the unique MGC-to-oocyte paracrine ligand-receptor pairs found in S#1, S#3 and S#7 follicles. **C** Venn diagram illustrating the unique MGC-to-oocyte paracrine ligand-receptor pairs found in S#2, S#8 and S#9 follicles. **D** Dot plot showing the interaction scores of specific MGC-to-oocyte paracrine ligand-receptor pairs in healthy small follicles and other small follicles. The plot highlights the consistency of interaction scores in healthy follicles compared to the heterogeneity observed in other follicles

Only a single dominant follicle can develop to ovulation in a bovine natural estrous cycle. This implies that antral follicles exhibit variability in their developmental states, even when they share uniform healthy morphological features at the time of sampling. Thus, we asked if the intrafollicular communications are heterogeneous among individual follicles. Focusing on MGC-to-oocyte interactions, we first examined three small follicles (S#4, S#5, S#6) that were transcriptomically defined as "healthy" (characterized by high *FSHR* expression, and low *TP53*/*FAS* expression). Venn diagram analysis across three small healthy follicle samples revealed a core set of shared ligand-receptor pairs (66.6%, 459/689), with each follicle having its own unique interaction pairs (Fig. 6A). Next, to understand how communication dysregulates during early atresia, we compared this healthy core network (the 459 shared interactions) against the interaction networks of the remaining morphologically normal but transcriptomically "unhealthy" small follicles (S#1, S#2, S#3, S#7, S#8, S#9). This comparison revealed two distinct dysregulation patterns: (1) aberrant activation of some interactions that were silenced in healthy follicles (Fig. 6B); (2) loss of core interactions that were present in healthy follicles, in combination with aberrant activation (Fig. 6C). Of note, several interaction pairs, such as FN1-PLAUR, COL1A1-ITGB1, COL1A2-ITGB1, IHH-HHIP, MDK-ITGB1 and ADM-RAMP2, are frequently activated in unhealthy follicles, suggesting their possible role in determining follicular fate, or their application as diagnostic markers or therapeutic targets. Another interesting finding is that the activity of core interactions tends to remain at a steady level in healthy follicles but show great variability among unhealthy follicles (Fig. 6D).

Similarly, in medium and large follicles, healthy samples shared a large proportion of core interactions while maintaining their own unique interactions. Unhealthy follicles exhibited a high incidence of abnormal activation of interactions that should be silent, or the loss of interactions that should be present in healthy follicles. (Fig. S8A, B).

## Discussion

Follicular development is a dynamic process driven by the "intercellular interactions-microenvironment adaptation-functional synergy", centered on the precise molecular crosstalk among MGCs, CCs, and the oocyte to balance developmental efficiency and fate decisions [46, 47]. Through transcriptome sequencing and ligand-receptor network analysis of these three cell types across different developmental stages of bovine antral follicles, we unveil a complex and multi-layered communication network, providing new insights into the molecular mechanisms underlying follicular development in mammals.

Our study redefines the signaling scope of the oocyte. Contrary to the conventional view that CCs, due to their close physical connection with the oocyte, are the primary recipients of oocyte-derived factors such as GDF9 and BMP15 [48], our data demonstrated that in bovine follicles, MGCs exhibit a relatively high representation and appear to be the principal targets of oocyte-derived signaling. The extensive number of inferred ligand-receptor pairs involved in MGC autocrine and oocyte-to-MGC paracrine communication, along with their consistent presence across developmental stages, suggests a potential role during follicular development. However, given that MGCs and oocytes are physically separated by cumulus cells, the zona pellucida, and follicular fluid, these inferred MGC-to-oocyte and oocyte-to-MGC interactions should be interpreted as predicted inter-compartment signaling relationships rather than confirmed direct physical contacts. It is highly probable that some of these signals operate indirectly via the cumulus cell relay.

Complementing these findings, we identified a crucial metabolic shift in CCs—a transition in energy metabolism from mitochondrial oxidative phosphorylation to glycolysis. Direct measurement of hypoxia within the follicular environment would strengthen the interpretation of this metabolic transition. However, accurate assessment of dissolved O₂ in follicular fluid remains technically challenging and may not reliably reflect the oxygen status experienced by cumulus cells. To complement GO-based analyses, we therefore incorporated additional transcriptome-derived approaches to evaluate hypoxic status, including HIF signaling activity scoring and enrichment analysis of cellular responses to hypoxia. These independent lines of evidence provide further support for the inferred metabolic shift in CCs. This shift may represent an adaptive response to the progressively established hypoxic microenvironment within the expanding antral follicle [49, 50]. The adaptive alteration in metabolic phenotype likely enables CCs to continuously supply energy and biosynthetic precursors (e.g., pyruvate) to the oocyte, thereby supporting its cytoplasmic maturation [51, 52]. Although previous studies reported glycolytic activity in CCs under in vitro conditions, no direct link had been established with the hypoxic follicular microenvironment in vivo [53]. This may partially explain the previous finding that pyruvate enhanced developmental competence of in vitro matured oocytes, especially for cumulus-denuded bovine oocytes [54], which not only elucidated the physiological adaptation of CC metabolic function but also identified a potential target for optimizing IVM systems.

Further analysis in this study revealed many-to-many interaction patterns in ligand-receptor interactions during antral follicular development, pointing toward a potential functional redundancy which may serve as a robust fail-safe mechanism. We hypothesize that this mechanism could safeguard the continuity of essential physiological processes—such as cell proliferation, adhesion, and survival—amid dynamic fluctuations in physiological conditions, thereby potentially preventing follicular developmental failure caused by the disruption of single signaling pathways [14, 55]. Most notably, the many-to-many ligand-binding pattern is observed in the integrin (ITG) family. Integrins are central mediators of cell–cell and cell–matrix interactions, yet their specific ligands [56, 57], downstream signaling pathways, and functional roles in the follicles remain poorly understood. By correlating ligand-receptor pairs with biological functions, we found that integrins and their ligands are highly expressed across various stages of follicular development and are involved in multiple fundamental processes such as cell adhesion and proliferation. This suggests that integrins may act as a key hub maintaining structural integrity and functional coordination within the follicular microenvironment. However, because true redundancy implies that the disruption of one pathway can be fully compensated by another, these concepts remain largely speculative at this stage. Future studies employing functional perturbation data—such as blocking specific integrin-ligand pairs—are required to validate these compensatory mechanisms.

To translate our transcriptomic map into practical applications, we functionally validated candidate MGC-derived ligands, notably PTN. While PTN supplementation during IVM did not yield a statistically significant increase in the overall blastocyst formation rate, it significantly enhanced the ICM/TE ratio. Because the ICM is the sole progenitor of the fetus, this improvement in cellular architecture is biologically highly meaningful, effectively bridging the developmental quality gap between in vitro-produced and in vivo-derived embryos. Mechanistically, our network predicted that PTN may interact with the PLXNB2 receptor and its downstream axon guidance pathway. Consistent with the classical role of this pathway in actin cytoskeleton remodeling, we demonstrated that PTN supplementation significantly increased the density of transzonal projections (TZPs)—the vital actin-rich physical conduits between CCs and the oocyte. These findings support the possibility that PTN improves aspects of oocyte maturation quality, at least in part, by promoting somatic-germ cell communication.

Furthermore, this study raises the intriguing possibility of a novel role of the oocyte in regulating immune-related pathways in MGCs. Although granulosa cells are known to possess innate immune functions [36, 37], the upstream mechanisms and signaling pathways controlling their immunoregulatory roles have remained elusive. Our data provided important transcriptomic clues that the oocyte may serve as a critical upstream regulator of this process. While functional validation is required, these predictions point to a potential new dimension of oocyte-granulosa cell communication: the oocyte may help maintain local immune tolerance within the follicle by modulating the immune function of granulosa cells, thereby protecting the avascular follicular structure from inflammatory damage, which is essential for follicular survival and healthy development [58, 59].

At the system level, this study reveals that follicular development is regulated by a spatiotemporal communication strategy exhibiting substantial commonality. Core ligand-receptor interactions appear to be extensively repurposed across different cellular pairs and developmental stages, forming a relatively stable core network. This evolutionary strategy might maximize signaling efficiency and support the robustness of follicular development, thereby potentially reducing the risk of developmental disorders caused by excessive regulatory complexity [60].

**Fig. 7:**
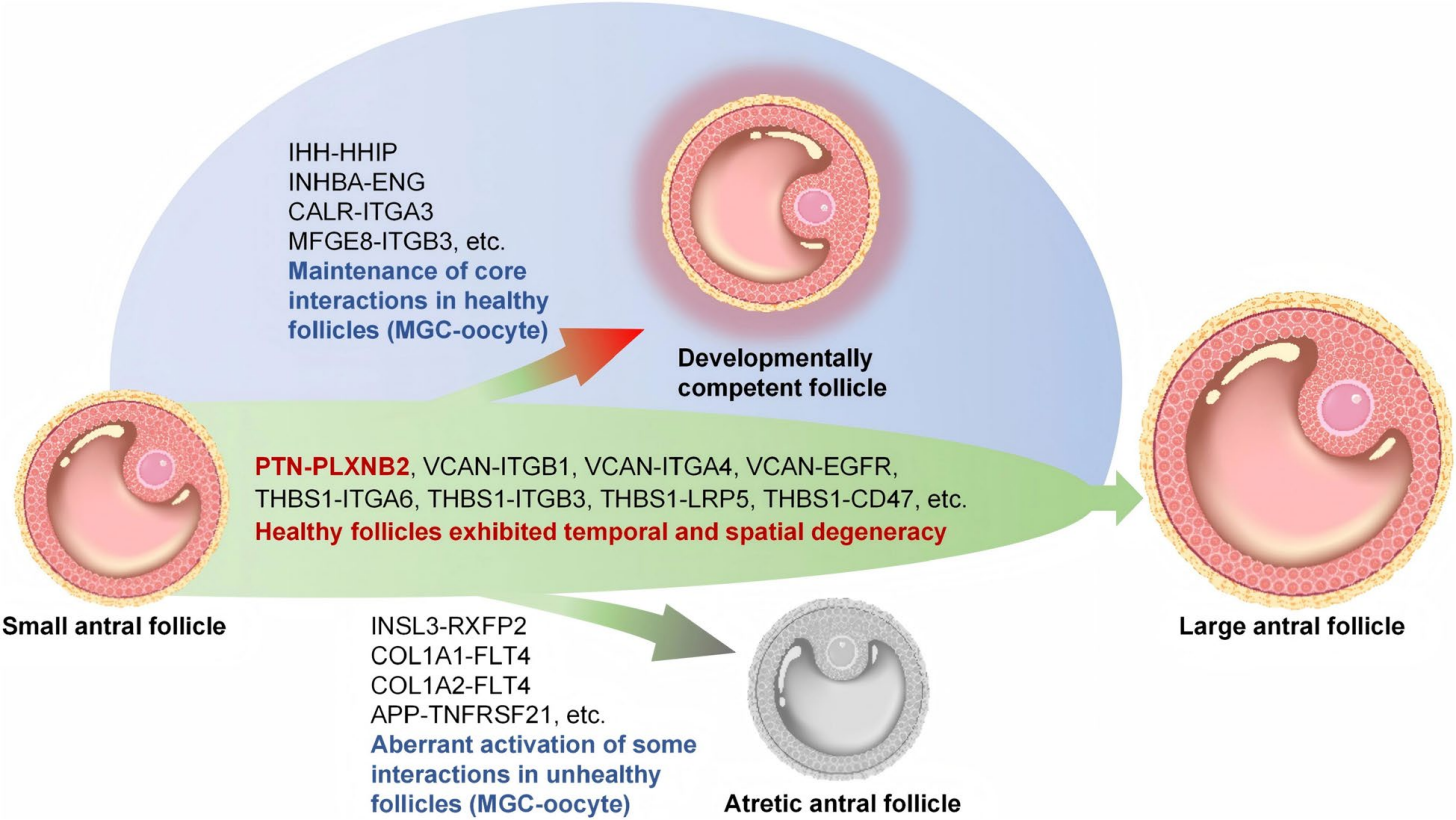
A dual-layer regulatory model of follicular development and fate specification. The first layer (core network) is characterized by extensive repurposing and commonality, ensuring the robustness and basic progression of follicular development. The second layer is a dynamic, context-specific tier that fine-tunes interactions to specify the ultimate follicle fate (ovulation or atresia). Unhealthy follicles exhibit network dysfunction through aberrant pathway activation or failure of essential communication connections

It should also be emphasized that the ultimate fate of a follicle (ovulation or atresia) appears not to be determined solely by the core network described above, but also relies on precise fine-tuning of an individualized interaction layer. Based on this, we propose a dual-layer regulatory model: the first layer consists of a core network characterized by high commonality and overlap that supports basic follicular developmental functions. This could allow follicles to rapidly initiate critical processes at different stages while avoiding developmental disorders caused by drastic reprogramming of gene expression profiles. This may also explain why the core mechanisms of follicular development are highly conserved across mammals [61, 62]. The second layer is a dynamic and context-specific regulatory tier, which may contribute to follicle fate specification—i.e., determining whether a follicle proceeds to ovulation or atresia. This model effectively accounts for the observed heterogeneity among follicles [36, 62]. Notably, we observed two distinct patterns of network dysfunction in unhealthy follicles: first, abnormal enrichment of non-physiological pathways, and second, reduced representation of essential communication connections. These transcriptome-derived changes in interaction networks may constitute a novel class of potential biomarkers for predicting developmental competence, offering potential diagnostic avenues in reproductive medicine (Fig. 7). It should be noted that this model is primarily based on transcriptomic inference, and additional functional studies will be required to validate the proposed regulatory mechanisms and their biological significance.

Finally, several inherent limitations of our study must be acknowledged. First, the interactome provided here is fundamentally predictive. Discrepancies between our interaction list and those from other established databases (e.g., CellChat) may arise from differences in primary data sources, scoring algorithms, and orthology mapping. Second, maternal mRNAs in the oocyte are often synthesized and stored for extended periods; thus, transcript detection does not definitively equate to contemporaneous protein translation or functional activation. Although we integrated proteomic data to verify the abundance of select candidate receptors (e.g., PLXNB2 and SDC3), the broader interaction map should be viewed as a repository of putative signaling candidates that warrant further targeted protein-level and functional validation.

## Conclusion

This study systematically elucidates the core principles of cellular interactions during bovine follicular development: MGCs exhibit a high representation in signal communication networks, while the oocyte appears to act as a critical signaling source with potential roles, including immunoregulatory interactions with MGCs. Follicular cells employ ligand-receptor strategies characterized by substantial commonality and overlap, which may contribute to communication robustness. Our findings demonstrate that follicular health is closely associated with the precise regulation of communication networks, and that alterations in specific interactions may be linked to follicular dysfunction. Our constructed intrafollicular interaction map can predict MGC-derived ligands that may enhance the developmental competence of oocytes after IVM. These discoveries not only broaden the understanding of follicular intercellular crosstalk and refine the transcriptome-based interaction network underlying follicular development in mammals, but also provide a theoretical foundation and potential technical targets for improving the efficiency of assisted reproductive technologies (ART) in both livestock and humans.

## Supplementary Information

Additional file 1: Fig. S1. Sample collection of follicular cell and quality control of transcriptome data. Fig. S2. Mfuzz analysis of gene expression on MGCs, CCs and oocytes and GO analysis of CCs and oocytes. Fig. S3. Sensitivity analysis of ligand-receptor expression and interaction-score thresholds. Fig. S4. Heatmap showing the interaction scores of 160 ligand-receptor pairs that are present in all six paracrine interaction types and across all three stages of follicular development. Fig. S5. Sankey diagram, expression of VCAN and ICAM4 and their effects on MGCs cell viability. Fig. S6. Sankey diagram and expression of JAG1 and PTN. Fig. S7. Overview of oocyte receptor expression and PTN-mediated intrafollicular communication dynamics. Fig. S8. MGC-oocyte paracrine interactions in a single follicle, medium follicles, large follicles.

Additional file 2: Table S1. RNA-seq data quality and sample exclusion guidelines. Table S2. Ligand expression in all individual follicles of three cell types. Table S3. Receptor expression in all individual follicles of three cell types. Table S4. Interaction scores of 1831 pairs in various interaction relationships. Table S5. DEG of small and medium follicles MGCs. Table S6. DEG of medium and large follicles MGCs. Table S7. DEG of small and medium follicles CCs. Table S8. DEG of medium and large follicles CCs. Table S9. DEG of small and medium follicles oocytes. Table S10. DEG of medium and large follicles oocytes. Table S11. KEGG enrichment analysis of small follicles specifically highly expressed genes on MGCs. Table S12. KEGG enrichment analysis of medium follicles specifically highly expressed genes on MGCs. Table S13. KEGG enrichment analysis of large follicles specifically highly expressed genes on MGCs. Table S14. KEGG enrichment analysis of small follicles specifically highly expressed genes on CCs. Table S15. KEGG enrichment analysis of medium follicles specifically highly expressed genes on CCs. Table S16. KEGG enrichment analysis of large follicles specifically highly expressed genes on CCs. Table S17. KEGG enrichment analysis of small follicles specifically highly expressed genes on oocytes. Table S18. KEGG enrichment analysis of medium follicles specifically highly expressed genes on oocytes. Table S19. KEGG enrichment analysis of large follicles specifically highly expressed genes on oocytes.

## Data Availability

All data analyzed during this study are available in the article and/or supporting information, further inquiries can be directed to the corresponding author on reasonable request. RNA-Seq data were deposited into the Gene Expression Omnibus database under accession number GSE330668.

## Abbreviations

ACVR2A: Activin A receptor type 2A
ADM: Adrenomedullin
ALK: ALK receptor tyrosine kinase
APOE: Apolipoprotein E
BMP15: Bone morphogenetic protein 15
BSA: Bovine serum albumin
CALCRL: Calcitonin receptor like receptor
CC: Cumulus cell
cGMP: Cyclic guanosine monophosphate
COC: Cumulus-oocyte complex
COL1A1: Collagen type I alpha 1 chain
DEG: Differentially expressed gene
EGFR: Epidermal growth factor receptor
FBS: Fetal bovine serum
FN1: Fibronectin 1
FSHR: Follicle stimulating hormone receptor
FST: Follistatin
GDF9: Growth differentiation factor 9
GSEA: Gene set enrichment analysis
HBEGF: Heparin-binding EGF-like growth factor
HHIP: Hedgehog interacting protein
ICAM4: Intercellular adhesion molecule 4
ICM: Inner cell mass
IHH: Indian hedgehog signaling molecule
INHA: Inhibin subunit alpha
ITG: Integrin
ITGA2B: Integrin subunit alpha 2B
ITGB1: Integrin subunit beta 1
IVM: In vitro maturation
JAG1: Jagged canonical notch ligand 1
LRP1/2/5: LDL receptor related protein 1/2/5
MDK: Midkine
MGC: Mural granulosa cell
NODAL: Nodal homolog
PCA: Principal component analysis
PDGFRB: Platelet derived growth factor receptor beta
PLXNB2: Plexin B2
PTN: Pleiotrophin
PTPRS: Protein tyrosine phosphatase receptor type S
PVA-PBS: Polyvinyl alcohol-phosphate-buffered saline
RAMP: Receptor activity modifying protein
SDC3: Syndecan 3
TE: Trophectoderm
TGF-beta: Transforming growth factor beta
THBS1: Thrombospondin 1
TPM: Transcripts per million
VCAN: Versican
VWF: Von willebrand factor
