# Supplementary material for: A transcriptomic map of ligand-receptor interactions within bovine antral follicles predicts intrafollicular factors for improving oocyte quality
Source: J Anim Sci Biotechnol. 2026 Jul 14;17:147. doi: 10.1186/s40104-026-01469-w (PMC13366956; doi:10.1186/s40104-026-01469-w)
Supplement: Supplementary file 1 — Additional file 1: Fig. S1. Sample collection of follicular cell and quality control of transcriptome data. Fig. S2. Mfuzz analysis of gene expression on MGCs, CCs and oocytes and GO analysis of CCs and oocytes. Fig. S3. Sensitivity analysis of ligand-receptor expression and interaction-score thresholds. Fig. S4. Heatmap showing the interaction scores of 160 ligand-receptor pairs that are present in all six paracrine interaction types and across all three stages of follicular development. Fig. S5. Sankey diagram, expression of VCAN and ICAM4 and their effects on MGCs cell viability. Fig. S6. Sankey diagram and expression of JAG1 and PTN. Fig. S7. Overview of oocyte receptor expression and PTN-mediated intrafollicular communication dynamics. Fig. S8. MGC-oocyte paracrine interactions in a single follicle, medium follicles, large follicles. [file 40104_2026_1469_MOESM1_ESM.pdf]

Additional file 1:

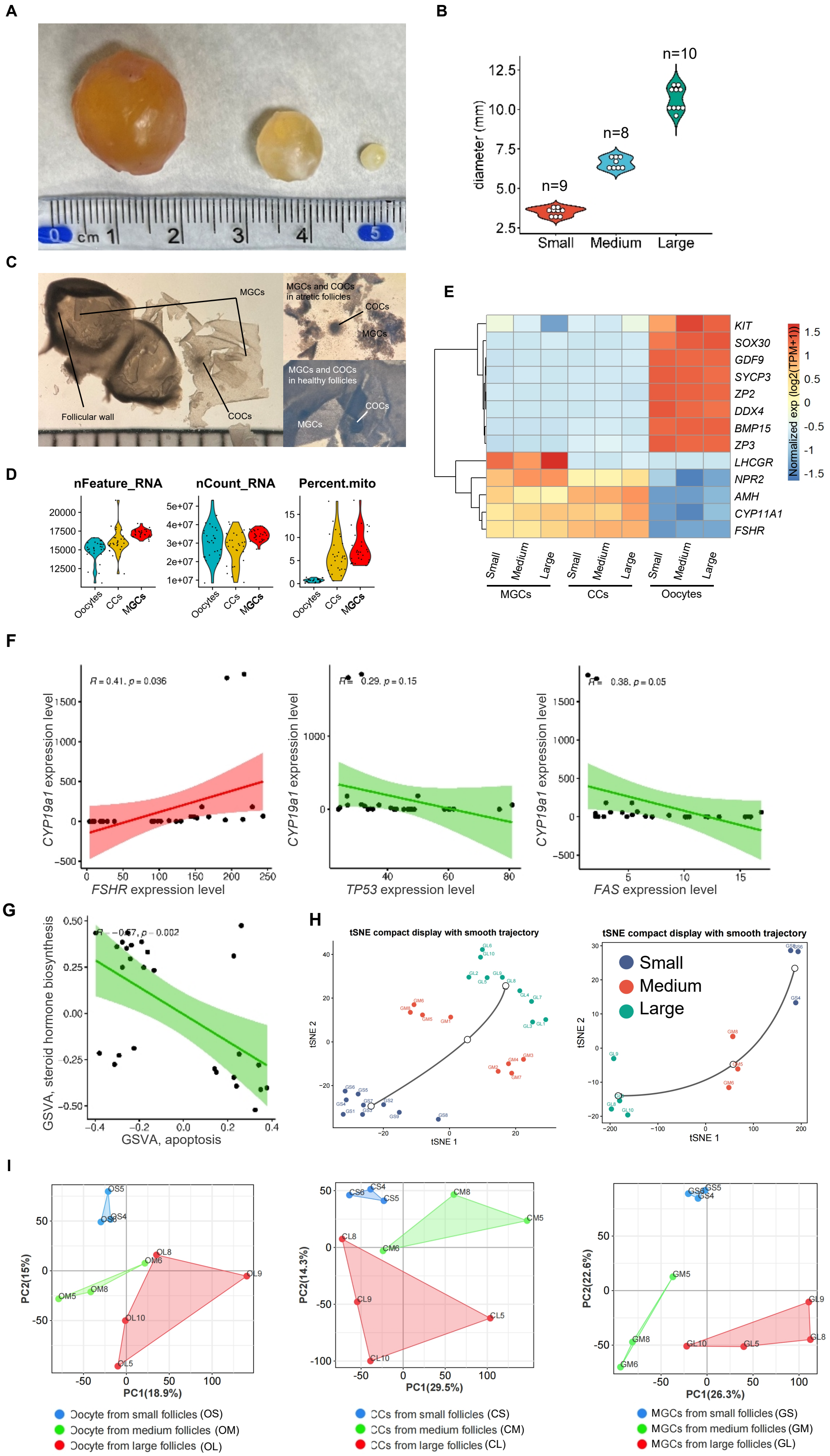

Fig. S1. Sample collection of follicular cell and quality control of transcriptome data. **A-B** Representative photos of large, medium, and small follicles (A) and the diameter statistics of each follicle (B). **C** Healthy versus atretic follicles were distinguished by gross morphology during follicle wall incision and collection of MGCs, CCs, and oocytes. **D** Violin plots showing the distributions of detected gene number (nFeature_RNA), total transcript/read count (nCount_RNA), and mitochondrial transcript proportion (percent.mito) in oocytes, CCs, and MGCs. Each dot represents one sample. The oocyte from follicle 71804 displayed an abnormally high mitochondrial transcript proportion and was excluded from downstream analysis; its matched MGC and CC samples were also removed to preserve the single-follicle matched design. **E** Heatmaps showing the expression of known marker genes in MGCs, CCs, and oocytes. **F-G** Correlation analysis of gene expression and pathway activities in MGCs. In all plots, each dot represents the MGCs derived from a single follicle. (F) Scatter plots showing the correlations between the expression level of CYP19A1 and FSHR, TP53, and FAS. (G) Scatter plot illustrating the correlation between the activities of the steroid hormone biosynthesis pathway and the apoptosis pathway, as determined by GSVA scores. **H** Pseudotime analysis using transcriptome data of MGCs from all follicles and healthy follicles. **I** PCA (2D) of RNA-seq data from bovine MGCs, CCs, and oocytes collected across follicle diameters. Samples were pre-screened by Fig. 1C-D.

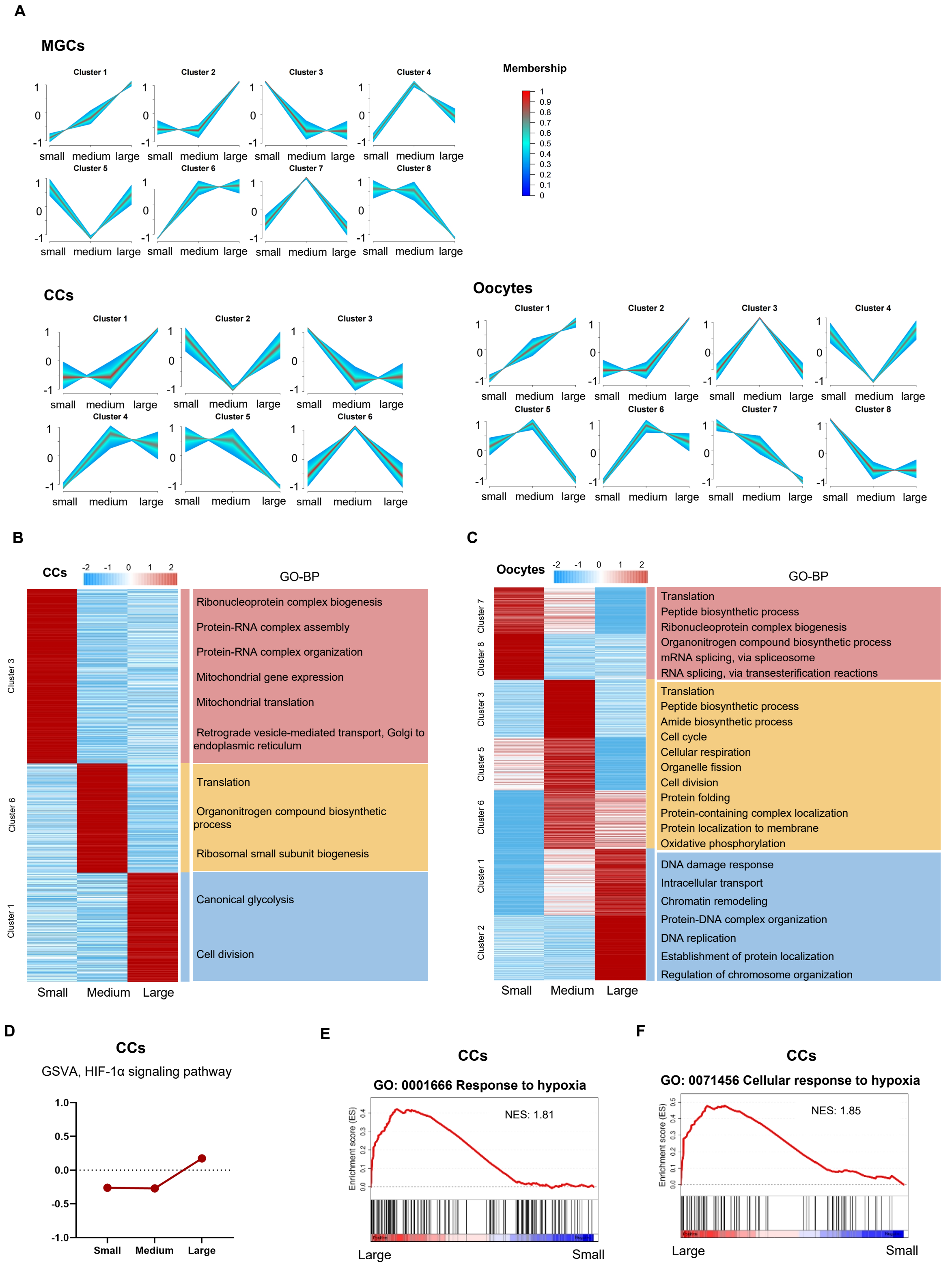

Fig. S2. Mfuzz analysis of gene expression on MGCs, CCs and oocytes and GO analysis of CCs and oocytes. **A** Mfuzz analysis of MGCs, CCs, and oocytes. **B-C** Heatmap of stage-specific high-expression genes in CCs (B) and oocytes (C) and corresponding GO-BP enrichment. **D** Analysis of HIF-1 Signaling Pathway GS Activity in CCs. **E-F** GSEA of dynamic changes in response to hypoxia (E) and cellular response to hypoxia (F) in CCs.

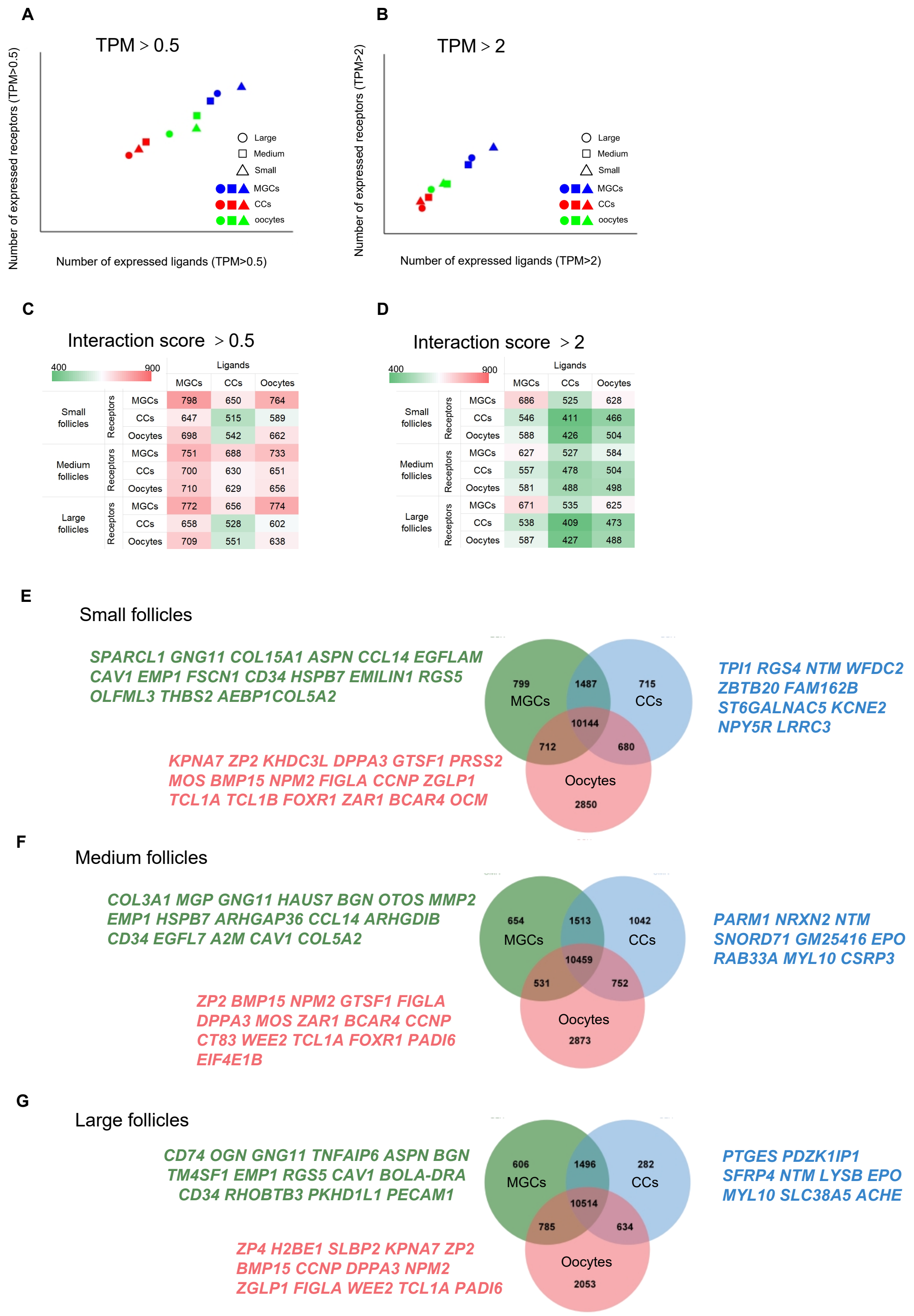

Fig. S3. Sensitivity analysis of ligand-receptor expression and interaction-score thresholds. **A- B** Scatter plot showing the number of ligands and receptors as coordinates for three types of cells (MGCs, CCs, and oocytes) across three stages of follicular development. **C-D** Heatmap showing the number of ligand-receptor pairs between three types of cells (MGCs, CCs, and oocytes) across three stages of follicular development. **E-G** Number of co-expressed and unique genes (TPM > 1) among MGCs, CCs, and oocytes in small follicles (E), medium follicles (F) and large follicles (G).

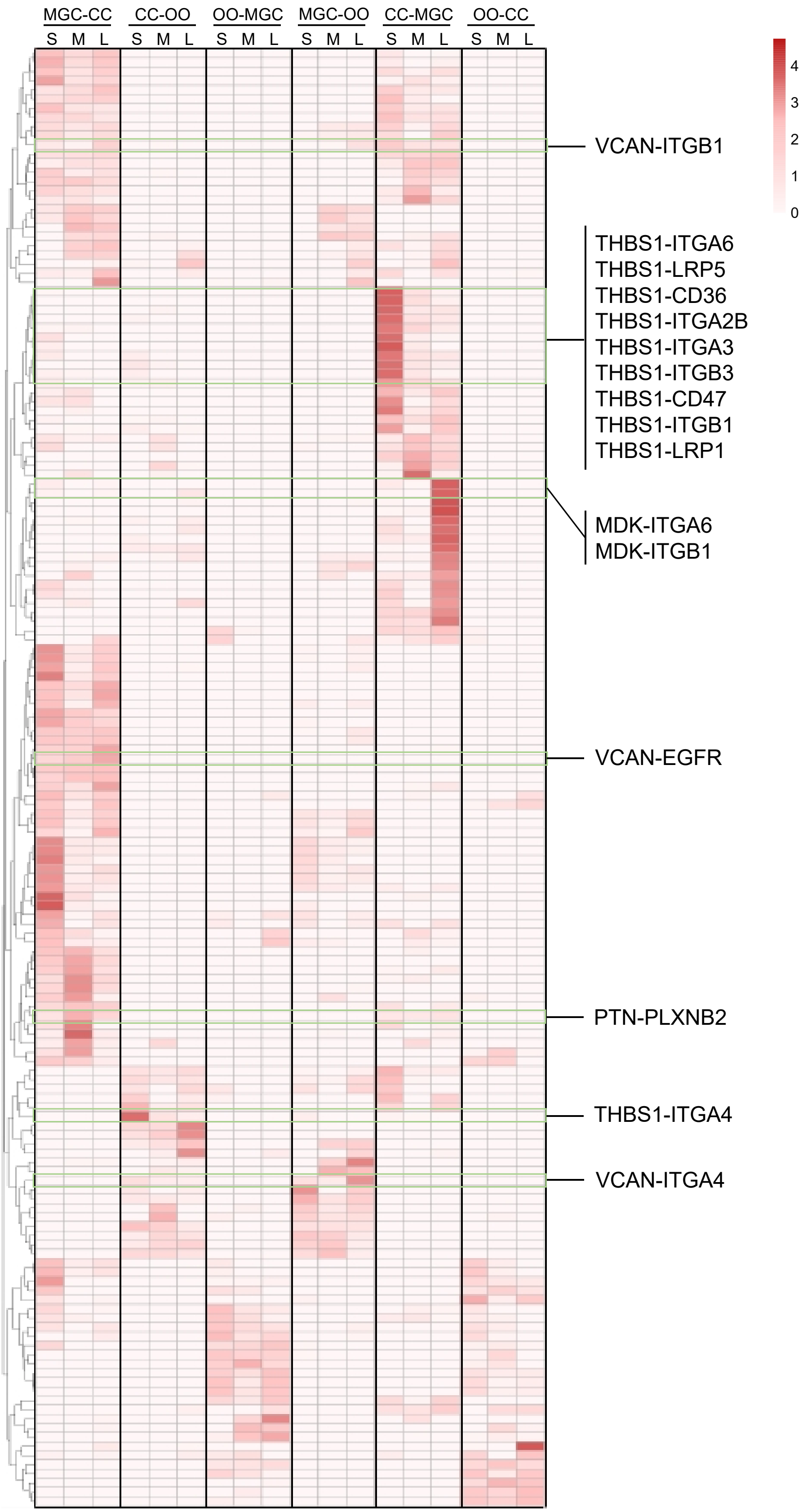

Fig. S4. Heatmap showing the interaction scores of 160 ligand-receptor pairs that are present in all six paracrine interaction types and across all three stages of follicular development (identified in Fig. 2G).

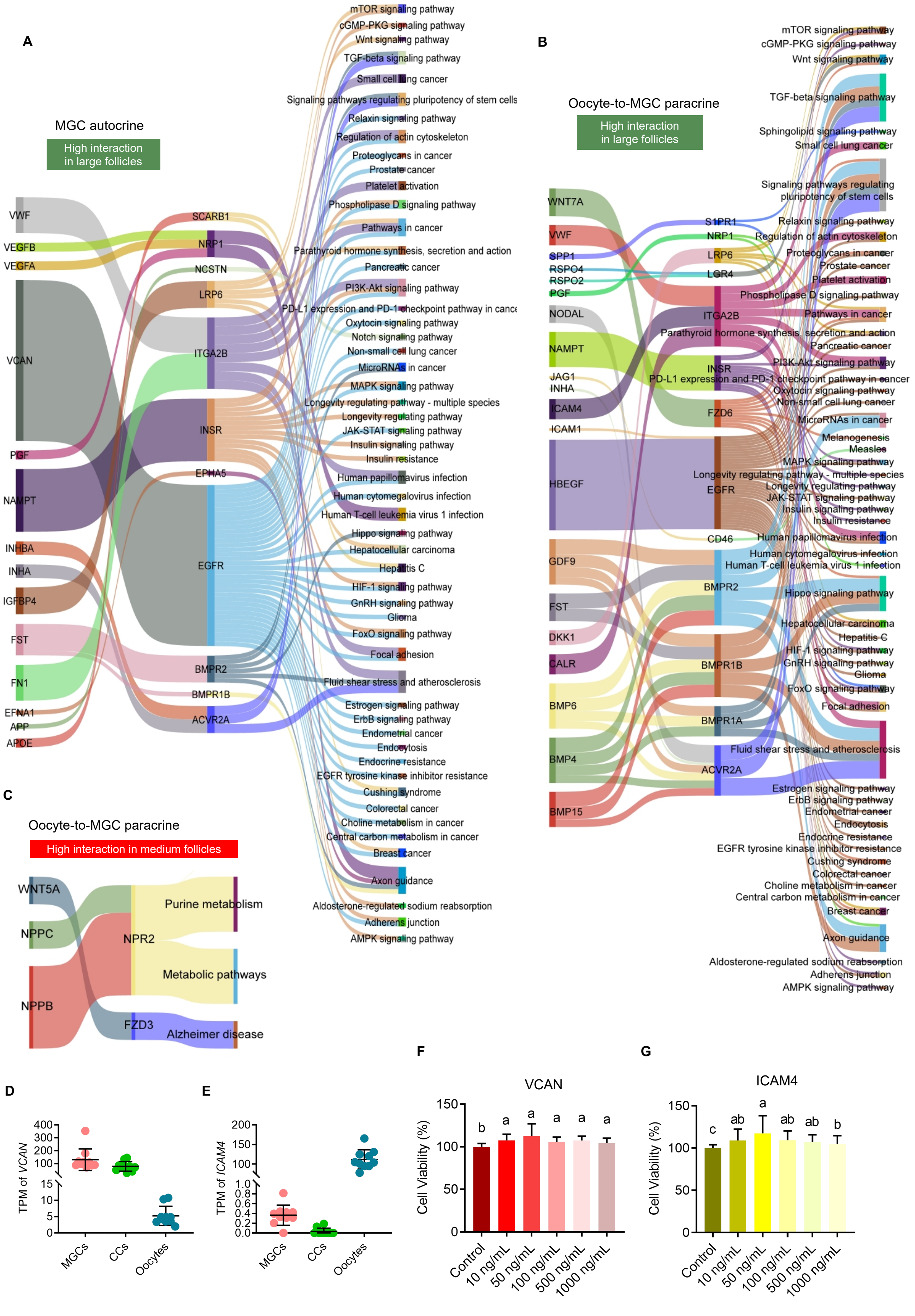
A
MGC autocrine
High interaction in large follicles
VWF
VEGFB
VEGFA
VCAN
PGF
NAMPT
INHBA
INHA
IGFBP4
FST
FN1
EFNA1
APP
APOE
SCARB1
NRP1
NCSTN
LRP6
ITGA2B
INSR
EPHA5
EGFR
BMPR2
BMPR1B
ACVR2A
mTOR signaling pathway
cGMP-PKG signaling pathway
Wnt signaling pathway
TGF-beta signaling pathway
Small cell lung cancer
Signaling pathways regulating pluripotency of stem cells
Relaxin signaling pathway
Regulation of actin cytoskeleton
Proteoglycans in cancer
Prostate cancer
Platelet activation
Phospholipase D signaling pathway
Pathways in cancer
Parathyroid hormone synthesis, secretion and action
Pancreatic cancer
PI3K-Akt signaling pathway
PD-L1 expression and PD-1 checkpoint pathway in cance
Oxytocin signaling pathway
Notch signaling pathway
Non-small cell lung cancer
MicroRNAs in cancer
MAPK signaling pathway
Longevity regulating pathway - multiple species
Longevity regulating pathway
JAK-STAT signaling pathway
Insulin signaling pathway
Insulin resistance
Human papillomavirus infection
Human cytomegalovirus infection
Human T-cell leukemia virus 1 infection
Hippo signaling pathway
Hepatocellular carcinoma
Hepatitis C
HIF-1 signaling pathway
GnRH signaling pathway
Glioma
FoxO signaling pathway
Focal adhesion
Fluid shear stress and atherosclerosis
Estrogen signaling pathway
ErbB signaling pathway
Endometrial cancer
Endocytosis
Endocrine resistance
EGFR tyrosine kinase inhibitor resistance
Cushing syndrome
Colorectal cancer
Choline metabolism in cancer
Central carbon metabolism in cancer
Breast cancer
Axon guidance
Aldosterone-regulated sodium reabsorption
Adherens junction
AMPK signaling pathway
B
Oocyte-to-MGC paracrine
High interaction in large follicles
WNT7A
VWF
SPP1
RSPO4
RSPO2
PGF
NODAL
NAMPT
JAG1
INHA
ICAM4
ICAM1
HBEGF
GDF9
FST
DKK1
CALR
BMP6
BMP4
BMP15
S1PR1
NRP1
LRP6
LGR4
ITGA2B
INSR
FZD6
EGFR
CD46
BMPR2
BMPR1B
BMPR1A
ACVR2A
mTOR signaling pathway
cGMP-PKG signaling pathway
Wnt signaling pathway
TGF-beta signaling pathway
Sphingolipid signaling pathway
Small cell lung cancer
Signaling pathways regulating pluripotency of stem cells
Relaxin signaling pathway
Regulation of actin cytoskeleton
Proteoglycans in cancer
Prostate cancer
Platelet activation
Phospholipase D signaling pathway
Pathways in cancer
Parathyroid hormone synthesis, secretion and action
Pancreatic cancer
PI3K-Akt signaling pathway
PD-L1 expression and PD-1 checkpoint pathway in cancer
Oxytocin signaling pathway
Non-small cell lung cancer
MicroRNAs in cancer
Melanogenesis
Measles
MAPK signaling pathway
Longevity regulating pathway - multiple species
Longevity regulating pathway
JAK-STAT signaling pathway
Insulin signaling pathway
Insulin resistance
Human papillomavirus infection
Human cytomegalovirus infection
Human T-cell leukemia virus 1 infection
Hippo signaling pathway
Hepatocellular carcinoma
Hepatitis C
HIF-1 signaling pathway
GnRH signaling pathway
Glioma
FoxO signaling pathway
Focal adhesion
Fluid shear stress and atherosclerosis
Estrogen signaling pathway
ErbB signaling pathway
Endometrial cancer
Endocytosis
Endocrine resistance
EGFR tyrosine kinase inhibitor resistance
Cushing syndrome
Colorectal cancer
Choline metabolism in cancer
Central carbon metabolism in cancer
Breast cancer
Axon guidance
Aldosterone-regulated sodium reabsorption
Adherens junction
AMPK signaling pathway
C
Oocyte-to-MGC paracrine
High interaction in medium follicles
WNT5A
NPPC
NPPB
NPR2
FZD3
Purine metabolism
Metabolic pathways
Alzheimer disease
D
TPM of VCAN
400
300
200
100
15
10
5
0
MGCs
CCs
Oocytes
E
TPM of ICAM4
200
150
100
1.0
0.8
0.6
0.4
0.2
0.0
MGCs
CCs
Oocytes
F
VCAN
Cell Viability (%)
150
100
50
0
b
a
a
a
a
a
Control
10 ng/mL
50 ng/mL
100 ng/mL
500 ng/mL
1000 ng/mL
G
ICAM4
Cell Viability (%)
150
100
50
0
c
ab
a
ab
ab
b
Control
10 ng/mL
50 ng/mL
100 ng/mL
500 ng/mL
1000 ng/mL

Fig. S5. Sankey diagram, expression of VCAN and ICAM4 and their effects on MGCs cell viability. **A-B** Sankey diagram illustrating the ligand-receptor-pathway relationships in MGC autocrine (A) and oocyte-MGC paracrine (B) interactions within the large follicle stage. **C** Sankey diagram illustrating the ligand-receptor-pathway relationships in oocyte-MGC paracrine interactions within the medium follicle stage. **D-E** Expression of the VCAN (D) and ICAM4 (E) in all follicles. **F-G** Cell viability assay of MGCs cultured with different concentrations of VCAN (F) and ICAM4 (G) factor.

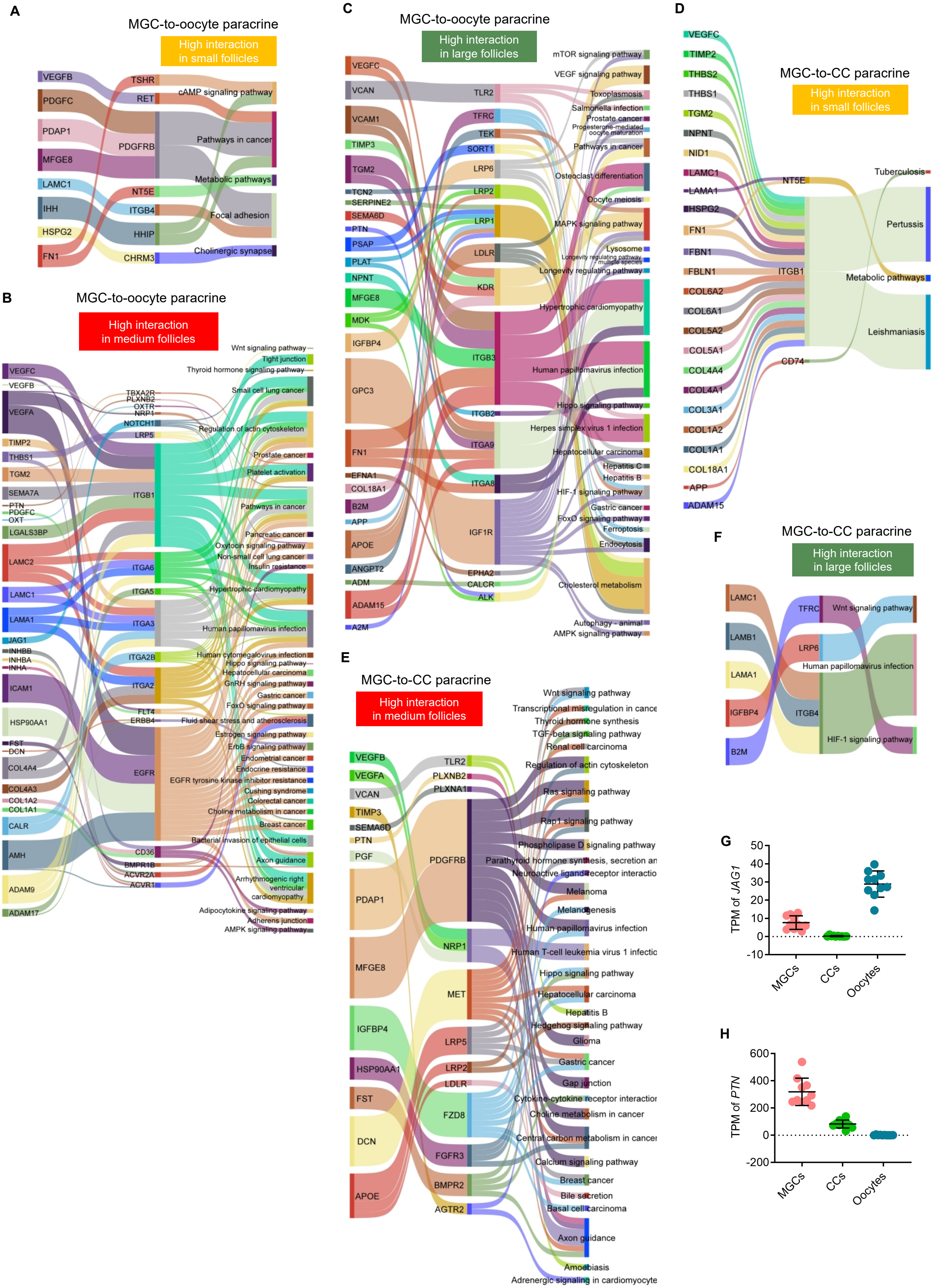

A
MGC-to-oocyte paracrine
High interaction in small follicles
VEGFB
PDGFC
PDAP1
MFGE8
LAMC1
IHH
HSPG2
FN1
TSHR
RET
PDGFRB
NT5E
ITGB4
HHIP
CHRM3
cAMP signaling pathway
Pathways in cancer
Metabolic pathways
Focal adhesion
Cholinergic synapse
B
MGC-to-oocyte paracrine
High interaction in medium follicles
C
MGC-to-oocyte paracrine
High interaction in large follicles
D
MGC-to-CC paracrine
High interaction in small follicles
E
MGC-to-CC paracrine
High interaction in medium follicles
F
MGC-to-CC paracrine
High interaction in large follicles
LAMC1
LAMB1
LAMA1
IGFBP4
B2M
TFRC
LRP6
ITGB4
Wnt signaling pathway
Human papillomavirus infection
HIF-1 signaling pathway
G
TPM of JAG1
MGCs
CCs
Oocytes
H
TPM of PTN
MGCs
CCs
Oocytes

Fig. S6. Sankey diagram and expression of JAG1 and PTN. **A-C** Sankey diagrams showing predicted ligand-receptor-pathway relationships for MGC-to-oocyte paracrine signaling during antral follicle development, including small (A), medium (B), and large (C) follicle stages. **D-F** Sankey diagrams showing predicted ligand-receptor-pathway relationships for MGC-to-CC paracrine signaling during antral follicle development, including small (D), medium (E), and large (F) follicle stages. **G-H** Scatter plots showing the expression patterns of JAG1 (G) and PTN (H) among MGCs, CCs, and oocytes, supporting their cell-type-enriched expression profiles. Data are presented as individual points with the mean±SEM.

A

The receptor on oocyte (receiver)

	Transcriptome		Proteome
High receptors	CD81	634.697	19.9726
	GPC4	106.298	13.2842
	ITGB1	86.5476	16.2240
	FLT1	47.0771	0.1184
	KIT	33.8538	1.9211
Medium receptors	SDC3	17.4293	2.32411
	ITGB4	10.8695	5.65285
	IGF2R	9.64605	6.37978
	IGF1R	7.10159	1.30652
	ITGA6	5.63883	10.3066
	EGFR	5.60673	1.84959
	NOTCH2	3.22097	1.77096
Low receptors	ITGA3	0.88147	3.19751
	ITGAV	0.70682	3.17964
	ITGA5	0.46625	0.21714
	PLXNB2	0.13405	1.07454
	INSR	0.11634	0.40965

B

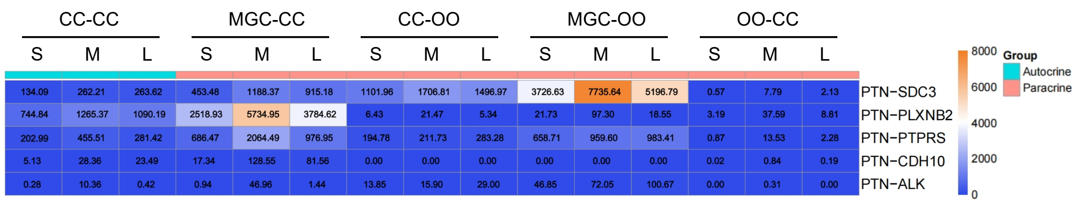

C

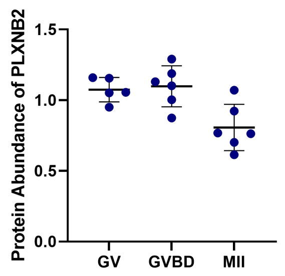

D

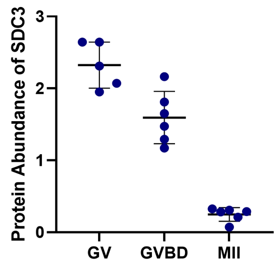

Fig. S7. Overview of oocyte receptor expression and PTN-mediated intrafollicular communication dynamics. **A** Comparative matrix summarizing selected receptors expressed in oocytes based on transcriptomic data and publicly available GV oocyte proteomic data. This panel provides a general overview of the oocyte receptor expression landscape and is not limited to PTN-associated receptors. **B** Heatmap illustrating the interaction scores of PTN and its corresponding receptors across different cell-cell communication axes during the small (S), medium (M), and large (L) stages of antral follicle development. The color gradient reflects the interaction strength, with blue indicating lower scores and orange/red indicating higher scores. The top annotation bars denote autocrine (cyan) and paracrine (pink) signaling modes. **C-D** Scatter dot plots showing the protein abundance of PTN receptors PLXNB2 (C) and SDC3 (D) in oocytes across sequential stages of meiotic maturation: GV, GVBD, and MII. Data are presented as individual data points with the mean ± SEM.

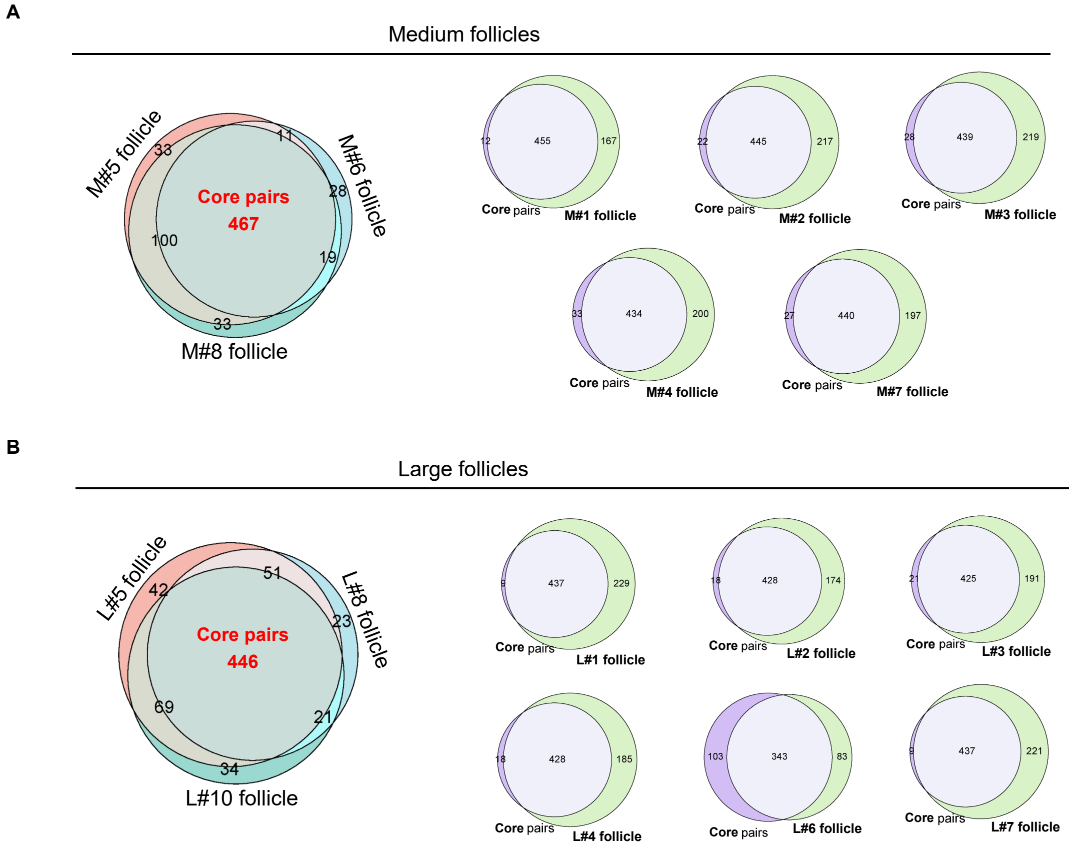

Fig. S8. MGC-oocyte paracrine interactions in a single follicle, medium follicles (A), large follicles (B).
